# Emotional problems and dysregulation in children/youth with ADHD: predictive and moderating effects in a randomized controlled trial of methylphenidate and neurofeedback

**DOI:** 10.1111/jcpp.70205

**Published:** 2026-07-28

**Authors:** Amelie Bohner, Hilario Blasco‐Fontecilla, Tomas Ros, Eric Acquaviva, Yohan Attal, Tobias Banaschewski, Sarah Baumeister, Elisa Bousquet, Aurore Bussalb, Marie Delhaye, Richard Delorme, Renate Drechsler, Allison Goujon, Alexander Häge, Louis Mayaud, Konstantin Mechler, Caroline Menache, Olivier Revol, Friederike Tagwerker, Susanne Walitza, Anna Maria Werling, Stéphanie Bioulac, Daniel Brandeis, Diane Purper‐Ouakil, Anna Kaiser

**Affiliations:** ^1^ Department of Child and Adolescent Psychiatry and Psychotherapy Central Institute of Mental Health, Medical Faculty Mannheim/Heidelberg University Mannheim Germany; ^2^ German Center for Mental Health (DZPG), Mannheim ‐ Heidelberg ‐ Ulm, partner site Mannheim Mannheim Germany; ^3^ Instituto de Investigación y Transferencia (ITEI), Universidad Internacional de La Rioja (UNIR), CIBERSAM Madrid Spain; ^4^ Department of Neuroscience Campus Biotech CISA‐Université de Genève Genève Switzerland; ^5^ Child and Adolescent Psychiatry Department and Child Brain Institute, Robert Debré Hospital Assistance Publique‐Hôpitaux de Paris and Universite Paris Cite Paris France; ^6^ Cyrebro Neurosignals Paris France; ^7^ Unit of Child and Adolescent Psychiatry (MPEA1), CHU Montpellier‐Saint Eloi Hospital University of Montpellier Montpellier France; ^8^ RDS SAS Strasbourg France; ^9^ Child and Adolescent Psychiatry, Erasme Academic Hospital Université Libre de Bruxelles Bruxelles Belgium; ^10^ Child and Adolescent Psychiatry and Psychotherapy, University Hospital of Psychiatry Zurich University of Zurich Zurich Switzerland; ^11^ Clinique des Grangettes Chêne‐Bougeries, Geneva Switzerland; ^12^ Unit of Child and Adolescent Psychiatry Hospices civils de Lyon, Hôpital Femme Mère Enfant Bron Cedex France; ^13^ Neuroscience Center Zurich University of Zurich and ETH Zurich Zurich Switzerland; ^14^ Zurich Center for Integrative Human Physiology University of Zurich Zurich Switzerland; ^15^ LPNC, UMR 5105 University Grenoble Alpes Grenoble France; ^16^ Unit of Child and Adolescent Psychiatry CHU Grenoble Alpes Grenoble France; ^17^ Development and Trajectories INSERM CESP U 1018 Psychiatry Montpellier France; ^18^ CESP, INSERM U 1018 Paul Brousse Hospital Villejuif France; ^19^ Department of Child and Adolescent Psychiatry/Psychotherapy University Hospital of Ulm, University of Ulm Ulm Germany

**Keywords:** emotional dysregulation, attention‐deficit/hyperactivity disorder, executive functions, neurofeedback, home treatment, methylphenidate

## Abstract

**Background:**

Children and adolescents with Attention‐Deficit/Hyperactivity Disorder (ADHD) often show increased emotional dysregulation (ED) and internalizing emotional problems (iEP). However, the predictive and moderating role of these emotional difficulties in the treatment of ADHD—particularly regarding pharmacological (methylphenidate, MPH) and non‐pharmacological approaches (neurofeedback at home, NF@home)—remains understudied. This study aims to evaluate the effectiveness of MPH and NF@home in reducing iEP/ED in children with ADHD and to assess the predictive role of iEP/ED and neuropsychological executive function (EF) measures in treatment outcomes.

**Methods:**

The per‐protocol sample included 146 children (7–13 years) diagnosed with ADHD (inattentive or combined type). iEP was measured with the Strengths and Difficulties Questionnaire (SDQ), ED via a dysregulation profile, and EF using the BRIEF and Continuous Performance Test (CPT). ADHD symptoms were assessed using the ADHD Rating Scale IV. Participants were randomized to either the MPH group (*n* = 59) or the NF group (*n* = 87), which included NF_SMR_ (*n* = 72) and NF_TBR_ (*n* = 15), based on individual Theta/Beta‐Ratio (TBR). Mixed‐model analyses considered symptom severity at baseline, age, gender, IQ, and study center as covariates.

**Results:**

All intervention groups showed significant changes in iEP, but with different effects regarding parent (NF_SMR_: *d* = −0.396, 95% CI [−0.574, −0.217]; NF_TBR_: *d* = −0.367, 95% CI [−0.747, 0.013]; MPH: *d* = −0.409, 95% CI [−0.603, −0.215]) and teacher ratings (NF_SMR_: *d* = −0.211, 95% CI [−0.405, −0.017]; NF_TBR_: *d* = −0.639, 95% CI [−1.06, −0.222]; MPH: *d* = −0.142, 95% CI [−0.340, 0.056]). There was no between‐intervention effect, supported by non‐inferiority analyses. For ED, we also found significant effects for all interventions; however, significantly more pronounced effects were obtained for MPH. Moreover, both prediction models indicated a limited predictive value.

**Conclusions:**

The findings suggest non‐inferiority of NF versus MPH treating iEP within an ADHD sample. However, due to limited predictive value, no clear clinical guidance can yet be drawn regarding personalized treatment recommendations. Further studies are needed to confirm and extend these findings.

## Background

With a global prevalence of about 5%, ADHD is one of the most common neurodevelopmental disorders with onset mainly in childhood (Polanczyk, de Lima, Horta, Biederman, & Rohde, 2007; Salari et al., 2023). However, ADHD is not limited to young age, as symptoms persist into adulthood in 40%–60% of cases (Faraone, Biederman, & Mick, 2006; Faraone et al., 2021), often with changes in symptom presentation and associated impairments (Faraone et al., 2006). It is characterized by inappropriate levels of inattention, hyperactivity, and impulsivity, and three presentations are distinguished: predominantly inattentive, predominantly hyperactive–impulsive, and combined (APA, 2013; WHO, 2019). In contrast to DSM‐5, ICD‐11 treats ADHD as a single diagnosis with variable symptom profiles (WHO, 2019), with the balance of inattention and hyperactivity–impulsivity changing across individuals and developmental stages.

Children with ADHD often show difficulties in regulating emotions (e.g., Anastopoulos et al., 2011). Empirical evidence confirms that emotional dysregulation (ED) frequently co‐occurs with ADHD (e.g., Banaschewski et al., 2012; Biederman et al., 2020; Galera et al., 2021). However, it remains debated whether ED represents a core ADHD symptom or a distinct construct influencing its expression (Anastopoulos et al., 2011; Shaw, Stringaris, Nigg, & Leibenluft, 2014). Conceptually, ED is a transdiagnostic feature that also occurs as a diagnostic criterion in other disorder categories (Anastopoulos et al., 2011; Astenvald, Frick, Neufeld, Bölte, & Isaksson, 2022; Hirsch, Chavanon, & Christiansen, 2019). It is characterized by inappropriate emotional reactivity, low frustration tolerance, and instability (Astenvald et al., 2022; Beheshti, Chavanon, & Christiansen, 2020; Shaw et al., 2014; Van Stralen, 2016). Multiple definitions and operationalizations exist (Deutz, Geeraerts, van Baar, Deković, & Prinzie, 2016; Freitag et al., 2023). Individuals with ADHD and concomitant ED are at higher risk for comorbid disorders and more severe social, motivational, and communicative impairments, as well as reduced quality of life (Ben‐Dor Cohen, Eldar, Maeir, & Nahum, 2021; Biederman et al., 2020; Galera et al., 2021; Van Stralen, 2016). Compared to ADHD without ED, they often exhibit more anger, impulsivity, and hyperactivity, and sometimes more inattention—likely contributing to poorer treatment outcomes besides the beforementioned (functional) difficulties (Caro‐Cañizares, Serrano‐Drozdowskyj, Pfang, Baca‐García, & Carballo, 2020).

ED can be expressed through internalizing and/or externalizing problems (Leibenluft et al., 2024). Especially during adolescence, internalizing emotional problems (iEP) become increasingly salient, particularly among females. In the literature, these are defined as difficulties that manifest in anxiety, shyness, avoidance, nervousness, fears, sadness, and worry and are characterized as “overly controlled” behaviors (Achenbach & Edelbrock, 1983; Armitage et al., 2023). Longitudinal studies show that early ADHD symptoms predict later iEP (Stern et al., 2020). Adolescents and young adults with comorbid ADHD and iEP display more severe ADHD symptoms (Bowen, Chavira, Bailey, Stein, & Stein, 2008), higher rates of psychiatric comorbidities (Bowen et al., 2008; Melegari et al., 2018), and poorer social functioning (Bishop, Mulraney, Rinehart, & Sciberras, 2019; Bowen et al., 2008).

Explanations for ED and iEP in ADHD include their emergence as consequences of chronic ADHD‐related impairments, their role as core ADHD features, or as factors leading to underdiagnosis in less overt cases (e.g., girls, migrants). Research suggests stronger associations between ED and hyperactivity–impulsivity (Groves, Kofler, Wells, Day, & Chan, 2020; Masi et al., 2020; Seymour, Chronis‐Tuscano, Iwamoto, Kurdziel, & MacPherson, 2014). Findings on iEP remain mixed, with links to both ADHD dimensions (Michelini, Eley, Gregory, & McAdams, 2015), but the association between hyperactivity–impulsivity and iEP appears to strengthen during the transition to young adulthood (Stern et al., 2020).

Neurobiological models propose shared dysfunctions underlying ADHD and emotional problems, particularly involving executive function (EF) deficits associated with the prefrontal cortex (Banaschewski et al., 2012). Children with ADHD perform worse on EF tasks than typically developing peers and medicated ADHD children, showing higher reaction times, more omission and go/no‐go errors, and poorer flexibility (Margari et al., 2016; Miklós Futó, Komáromy, & Balázs, 2019; Miranda et al., 2012). However, the link between iEP/ED and EF in ADHD remains unclear (Rabinovitz, O'Neill, Rajendran, & Halperin, 2016). Some findings indicate greater EF impairments in ADHD patients with higher ED levels (Biederman et al., 2020). Studies differentiating between externalizing and internalizing emotional problems and their relation to EF are still lacking.

Treatment of ADHD is assumed to be most effective when multimodal and adaptive (Becker et al., 2020; Döpfner et al., 2017; Geissler et al., 2018; Zinnow et al., 2018). Stimulants such as methylphenidate (MPH) are first‐line treatments for moderate to severe symptoms (Catalá‐López et al., 2017). Behavioral therapy (BT), cognitive training, neurofeedback (NF), and complementary approaches (e.g., diet, physical activity) might also be effective—especially in mild to moderate cases, as add‐ons, or when medication is not tolerated or preferred (Catalá‐López et al., 2017; Purper‐Ouakil et al., 2022). To train self‐regulation, NF uses EEG feedback by targeting brain activity patterns typical in ADHD (Clarke, Barry, McCarthy, Selikowitz, & Johnstone, 2007; Cortese et al., 2016). It aims to improve neuropsychological and behavioral self‐regulation (Catalá‐López et al., 2017; Enriquez‐Geppert, Smit, Pimenta, & Arns, 2019; Gevensleben, Moll, Rothenberger, & Heinrich, 2014). While some studies report (ADHD) symptom improvements (Aggensteiner et al., 2019; Bink, Van Nieuwenhuizen, Popma, Bongers, & Van Boxtel, 2014; Duric, Assmus, Gundersen, & Elgen, 2012), results remain inconsistent (Catalá‐López et al., 2017; Cortese et al., 2016). Meta‐analyses suggest that NF is promising but not (yet) as a stand‐alone treatment (Westwood et al., 2024). Home‐based NF might show additional potential due to higher accessibility, lower costs, and ecological validity (Bioulac et al., 2019; Kaiser et al., 2024; Purper‐Ouakil et al., 2022). In the NEWROFEED trial, both MPH and home‐based NF have been compared and results show that both treatments reduced ADHD core symptoms, but home‐based NF did not demonstrate non‐inferiority to MPH (Purper‐Ouakil et al., 2022).

Both, MPH and NF may also help reduce emotional problems, and some psychosocial interventions also explicitly target iEP and ED (e.g., Geissler, Vloet, Romanos, Zwanzger, & Jans, 2019), though robust evidence remains limited (Easdale‐Cheele, Parlatini, Cortese, & Bellato, 2024; Purper‐Ouakil et al., 2022; Suzer Gamli & Tahiroglu, 2018; Ventura et al., 2022; Winters, Fukui, Leibenluft, & Hulvershorn, 2018). However, improvements in emotional problems might be smaller than for core ADHD symptoms because all of these interventions mainly target core symptoms (Biederman et al., 2020). Higher baseline ED predicts greater ADHD severity and poorer treatment response over time (Karalunas, Gustafsson, Fair, Musser, & Nigg, 2019; Masi et al., 2020). Still, evidence on the moderating and predictive role of iEP/ED—differentiating between externalizing and internalizing emotional problems—in ADHD treatment outcomes taking into account innovative non‐pharmacological treatment alternatives remains limited (Fernández De La Cruz et al., 2015). Similarly, evidence regarding the predictive role of EF for iEP/ED is scarce; although, given that EF has not been identified as a reliable predictor of ADHD treatment outcomes following MPH or NF@Home interventions (Kaiser et al., 2024), EF might allow for predictions of treatment‐related changes in (associated) emotional problems. Therefore, this study aims to examine the effects of MPH and NF on ED/iEP in children and adolescents with ADHD. It also provides a contribution to the ongoing debate of the conceptual link between ADHD and internalizing as well as externalizing emotional problems and examines the role of emotional problems in ADHD‐focused treatment (outcomes). We expect higher ADHD symptom severity to correlate with greater ED and iEP severity, with stronger associations between ADHD and ED due to their higher conceptual overlap. We further hypothesize stronger links between ED and hyperactive–impulsive symptoms, and between iEP and inattentive symptoms. Treatment effects of MPH and home‐based NF will be compared: Both are expected to reduce iEP/ED (Aggensteiner et al., 2019; Golubchik, Rapaport, & Weizman, 2017; Suzer Gamli & Tahiroglu, 2018), as they are assumed to enhance prefrontal cortical top‐down processes and cognitive self‐control which may be associated, especially with improved ADHD core symptoms and ED (Chiu et al., 2022; Rubia et al., 2011; Suzer Gamli & Tahiroglu, 2018), with stronger effects anticipated for MPH and smaller effects on iEP. Furthermore, NF is expected to show sustained effects that may emerge with a delay, due to the learning principles and procedural learning mechanisms underlying NF (Van Doren et al., 2019). Reflecting a more complex symptomatology, higher baseline iEP/ED levels in ADHD are expected to predict smaller treatment gains (notwithstanding the greater potential for improvement associated with elevated baseline iEP/ED). To provide a more holistic assessment of the constructs, we included EF in our analyses as an indicator of higher cognitive functions alongside the behavioral markers of ADHD and iEP/ED. Considering the role of EF in ADHD and emotional problems (Biederman et al., 2020; Margari et al., 2016; Miklós et al., 2019; Miranda et al., 2012), we assume that greater EF impairments will be associated with higher iEP/ED severity but only small predictive effects for changes in iEP/ED following treatment (Kaiser et al., 2024).

## Methods

### Study design

In the randomized, multicenter NEWROFEED trial, 7‐ to 13‐year‐olds with ADHD received either MPH or NF@Home. Over 3 months, eight visits occurred, including group‐specific intervention sessions and evaluations at Day 0 (D0), D60, and D90. At D0, participants were randomized to either the MPH or the NF@Home group. Subsequently, participants attended four NF familiarization sessions or MPH titration visits until they were adequately trained in the use of the NF device or an optimal dose was achieved, respectively. Thereafter, NF sessions were conducted at home at a frequency of four sessions per week until the intermediate visit (D60), and this schedule was maintained until the final visit (D90) (Bioulac et al., 2019; Purper‐Ouakil et al., 2022). This study adheres to the CONSORT 2010 guidelines for the reporting of randomized controlled trials (CONSORT Statement).

### Trial registration

The trial was registered with the US National Institute of Health and is available online (https://clinicaltrials.gov/study/NCT02778360). The protocol was first subitted on 12 May 2016.

### Study sample

A priori power analysis set the target sample at 179 (allowing for 5% exclusion), recruiting 7–13‐year‐olds with inattentive or combined ADHD across nine centers in five European countries (Belgium, France, Germany, Spain, and Switzerland) between August 2016 and September 2017. Randomization (3:1) allocated 107 to NF@Home and 72 to MPH (see Figure 1). Analyses included per‐protocol (full participation) and mITT populations (minimum one session or MPH dose) (Bioulac et al., 2019; Purper‐Ouakil et al., 2022).

**Figure 1:**
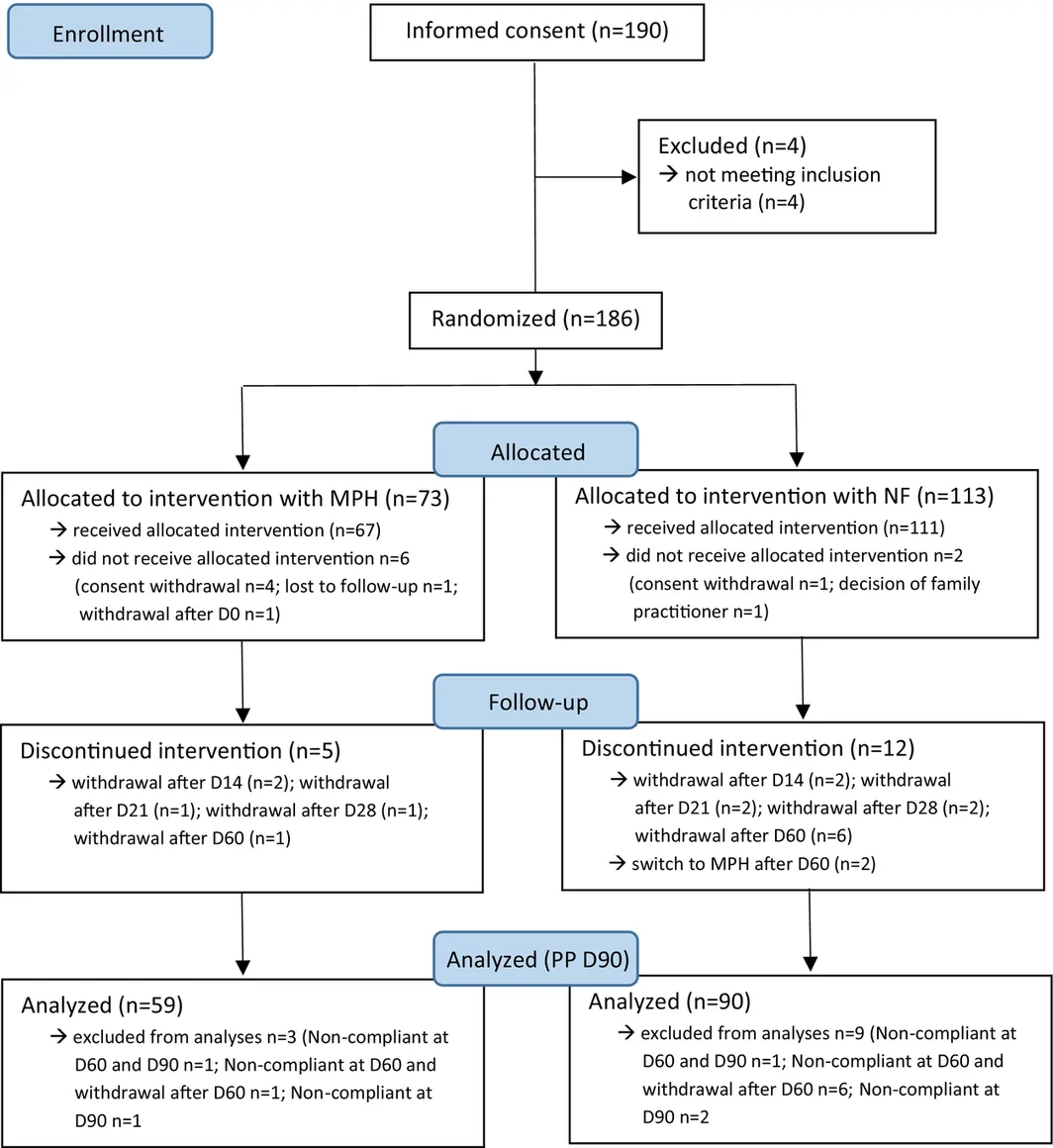
NEWROFEED CONSORT study flow chart (Purper‐Ouakil et al., 2022)

Eligibility required age 7–13, K‐SADS ADHD diagnosis, prior psychoeducation but no MPH or NF treatment, informed consent, and home internet access. Exclusion criteria: see Appendix S1.

### Study interventions

#### NF@Home group

A portable NF device (Mesia Koala™; Mensia Technologies) was used to apply individualized NF protocols depending on the individual qEEG profile. After the assessment of the individual alpha peak frequency, participants with a Theta/Beta Ratio (TBR) >4.5 μV received TBR down‐regulation training, whereas those with TBR <4.5 μV received sensorimotor rhythm (SMR) up‐regulation training, resulting in two subgroups: NF_TBR_ and NF_SMR_ (Purper‐Ouakil et al., 2022). Each training session consisted of five 4‐min active NF@Home blocks with immediate and real‐time feedback and two 2.5‐min transfer blocks with intermittent feedback. Following the device instruction at the initial visit (D0) up to four sessions, subsequent sessions were conducted at home (Bioulac et al., 2019).

#### Methylphenidate group

Medikinet® was used for pharmacological treatment starting with an initial dose of 10 mg/day, adjustable to 60 mg/day. After the beginning of the 2‐month treatment phase (D28), dosage remained stable (Purper‐Ouakil et al., 2022).

### Study outcomes

The primary outcome of the trial (Purper‐Ouakil et al., 2022) was the change in clinician‐rated ADHD score (ADHD Rating Scale IV; DuPaul, Power, Anastopoulos, & Reid, 1998) between D0 and D90 due to a treatment with MPH or NF@Home. Additionally, the study aimed to assess whether non‐inferiority of NF@Home compared with MPH could be demonstrated. As secondary analyses, the following variables and their intervention‐related changes were included in the analyses: (1) parent‐ and teacher‐rated ADHD RS IV total scores and subscales (inattentive, hyperactive–impulsive); (2) behavioral screening via the SDQ (Goodman, 1997); (3) executive functions assessed by the Behavior Rating Inventory of Executive Function (BRIEF; Mahone et al., 2002); and (4) attention performance measured with the Conners Continuous‐Performance Test 3rd Edition (Conners CPT 3; Conners, 2014). Additional secondary outcomes are detailed in the study protocol (Bioulac et al., 2019; Purper‐Ouakil et al., 2022). In contrast to these analyses, the primary outcome of the present study was the change in iEP/ED between D0 and D90 associated with treatment with MPH or NF@Home. Furthermore, a mixed‐effects prediction model was employed to examine whether EF predicts changes in iEP/ED following treatment with MPH or NF@Home, rather than relying on pre–post comparisons of primary and secondary outcomes.

### Operationalization and data analysis

Change scores between baseline (D0) and day 90 (D90) were used to assess changes in symptoms. As a brief behavioral screening questionnaire with robust psychometric properties (Goodman, 2001), the SDQ enables the simultaneous assessment of iEP (subscale “emotional problems”) and ED (“emotional problems,” “conduct problems,” “hyperactivity”) and was therefore employed in the present study. Baseline severity of iEP was assessed via parent and teacher ratings on the SDQ Emotional Problems subscale, capturing somatic complaints, worries, fears, and nervousness. ED was operationalized as the sum of the SDQ subscales Emotional Problems, Conduct Problems (e.g., temper tantrums, bullying, cheating, stealing), and Hyperactivity/Inattention (e.g., restlessness, limited concentration) at D0. Analyses included both iEP and ED, with change operationalized as parent‐ and teacher‐rated differences from D0 to D90. Neuropsychological indices of EF were measured using the BRIEF total score (parent‐rated EF) and several CPT scales (hit reaction time, variability, omission, and commission errors).

Data from the PP sample were analyzed using SPSS (IBM Corp., 2022) and R (R Core Team, 2022), with *α* = .05 and Bonferroni corrections for multiple testing where applicable. Pearson correlations examined associations between iEP/ED and ADHD symptoms at D0, including sub‐dimensions; dependent correlations were compared via Steiger's Z‐test. Correlations between neuropsychological EF indices and iEP/ED at D0 were also calculated.

ANCOVAs tested the effects of NF@Home versus MPH on iEP/ED change scores, controlling for baseline iEP/ED, gender, age, IQ, and study center. Here, we report the adjusted mean differences of the change scores (MD) resulting from the respective treatment conditions. Additionally, to assess non‐inferiority, margins were defined as 0.3 times the pooled standard deviation (*SD*) of change scores within the sample. Mean differences and 95% confidence intervals between treatments were calculated. Non‐inferiority was established if the upper confidence limit was below the *SD*‐based margin (Althunian, de Boer, Groenwold, & Klungel, 2017; Walker, 2019). Multiple linear regressions assessed moderating effects of baseline iEP/ED on treatment response (ADHD symptom reduction D0–D90), including main and interaction effects of treatment group and baseline iEP/ED, with covariates as above. A simple regression tested baseline ADHD symptoms as predictors of symptom change; a sensitivity analysis excluded baseline ADHD to confirm robustness.

Exploratory regressions evaluated the predictive value of neuropsychological EF indices on iEP/ED changes by treatment group, controlling for baseline iEP/ED and covariates. Sensitivity analyses tested robustness after omitting baseline iEP/ED.

## Results

### Sample description

The PP sample consisted of *N* = 146 children and adolescents with the combined or predominantly inattentive subtype of ADHD. 59 patients (15.3% female) were randomly assigned to the MPH group and 87 patients to the NF@Home group. Depending on the individual EEG‐patterns, there were *n* = 72 in the NF_SMR_ group (23.6% female) and *n* = 15 in the NF_TBR_ group (20.0% female). Further demographic data and descriptive statistics are shown in Appendix S2. There were no significant between‐group differences in sociodemographic variables or in baseline clinical, emotional, and neuropsychological characteristics.

### Correlations between iEP/ED and ADHD

As the SDQ was only assessed by parents and teachers, it should be mentioned in advance that clinician‐rated ADHD symptoms were correlated with parent‐rated iEP/ED. The correlations between clinician‐, parent‐, and teacher‐rated ADHD total score and parent‐ and teacher‐rated iEP/ED are listed in Appendices S3 and S4.

Firstly, the correlations between inattentive/hyperactive–impulsive symptoms and iEP/ED within each rater were compared. Clinician‐ratings showed descriptively stronger, positive correlations between the inattentive symptoms and iEP (*r* = .24, 95% CI [0.08, 0.39], *p* = .003) than for the hyperactive–impulsive symptoms and iEP (*r* = .14, 95% CI [−0.03, 0.29], *p* = .098). However, no significant differences were found (*Z* = −0.91, *p* = .364). With ED, stronger, positive correlations were found for hyperactive–impulsive symptoms (*r* = .53, 95% CI [0.40, 0.63], *p* = <.001) than for inattentive symptoms (*r* = .26, 95% CI [0.11, 0.41], *p* = .001) and they differed significantly (*Z* = 2.56, *p* = .015).

With regard to parent ratings, descriptively stronger positive correlations were observed between hyperactive–impulsive symptoms and iEP (*r* = .20, 95% CI [0.04, 0.36], *p* = .015) than between inattentive symptoms and iEP (*r* = .18, 95% CI [0.01, 0.33], *p* = .038), although this difference was not statistically significant (*Z* = −0.25, *p* = .804). A similar pattern emerged for ED (see Appendix S4); however, in this case, the correlations differed significantly (*Z* = −3.53, *p* < .001).

Teacher ratings, by contrast, showed descriptively stronger positive correlations between inattentive symptoms and iEP (*r* = .15, 95% CI [−0.04, 0.32], *p* = .116) than between hyperactive–impulsive symptoms and iEP (*r* = .03, 95% CI [−0.15, 0.21], *p* = .744), although this difference was likewise not statistically significant (*Z* = 0.82, *p* = .378). For ED, identical correlations were observed for inattentive and hyperactive–impulsive symptoms (*r* = .64, 95% CI [0.52, 0.74], *p* < .001), indicating no significant difference between them (*Z* = 0.04, *p* = .971).

### Within‐ and between‐group effects: iEP/ED symptom change D0–D90

To evaluate the within‐ and between‐group effects, the effect of each treatment (MPH, NF_SMR_, NF_TBR_) on iEP/ED was first assessed separately. Subsequently, comparisons across all treatment groups were conducted to determine between‐group effects.

Parent‐ratings showed a significant decrease in iEP between D0 and D90 for NF_SMR_ (MD = −1.01, *SE* = 0.23, 95% CI [−1.46, −0.55], *t*(134) = −4.38, *p* < .001, *d* = −0.396) and MPH (MD = −1.04, *SE* = 0.25, 95% CI [−1.54, −0.55], *t*(134) = −4.16, *p* < .001, *d* = −0.409). For NF_TBR_, there was a marginally non‐significant effect (MD = −0.94, *SE* = 0.49, 95% CI [−1.90, 0.03], *t*(134) = −1.91, *p* = .058, *d* = −0.367). To examine whether these decreases differed between the intervention groups, they were contrasted. There was no significant difference between NF_SMR_ and NF_TBR_ (MD = −0.07, *SE* = 0.55, 95% CI [−1.15, 1.01], *t*(134) = −0.13, *p* = .990, *d* = −0.029). Likewise, NF_SMR_ and MPH did not differ significantly (MD = 0.03, *SE* = 0.35, 95% CI [−0.65, 0.72], *t*(134) = .10, *p* = .995, *d* = 0.013) as well as contrasting NF_TBR_ and MPH (MD = 0.11, *SE* = 0.55, 95% CI [−0.98, 1.19], *t*(134) = 0.20, *p* = .979, *d* = 0.042).

Regarding the change in ED, parent‐ratings showed a significant decrease between D0 and D90 for NF_SMR_ (MD = −1.71, *SE* = 0.37, 95% CI [−2.45, −0.98], *t*(130) = −4.60, *p* < .001, *d* = −0.388) and MPH (MD = −1.57, *SE* = 0.41, 95% CI [−4.38, −2.77], *t*(130) = −8.77, *p* < .001, *d* = −0.801); however, NF_TBR_ did not reach a significant effect (MD = −0.47, *SE* = 0.76, 95% CI [−2.01, 1.07], *t*(130) = −0.61, *p* = .544, *d* = −0.104). Whereas NF_SMR_ and NF_TBR_ (MD = −1.24, *SE* = 0.87, 95% CI [−2.95, 0.47], *t*(130) = −1.43, *p* = .326, *d* = −0.284) did not differ significantly, both NF_SMR_ and MPH (MD = 1.86, *SE* = 0.57, 95% CI [0.74, 2.98], *t*(130) = 3.29, *p* = .004, *d* = 0.164) and NF_TBR_ and MPH (MD = 3.10, *SE* = 0.88, 95% CI [1.37, 4.84], *t*(130) = 3.54, *p* = .002, *d* = 0.308) differed significantly, both in favor of MPH.

Considering the teacher‐ratings, a significant decrease in iEP was observed for NF_SMR_ (MD = −0.51, *SE* = 0.24, 95% CI [−0.98, −0.04], *t*(82) = −2.17, *p* = .033, *d* = −0.211) and NF_TBR_ (MD = −1.54, *SE* = 0.50, 95% CI [−2.54, −0.54], *t*(82) = −3.05, *p* = .003, *d* = −0.639), whereas MPH revealed no significant effect (MD = −0.34, *SE* = 0.24, 95% CI [−0.82, 0.13], *t*(82) = −1.43, *p* = .157, *d* = −0.142). Contrasting the intervention groups, no significant differences were found comparing NF_SMR_ and NF_TBR_ (MD = 1.03, *SE* = 0.56, 95% CI [−0.09, 2.15], *t*(82) = 1.84, *p* = .164, *d* = 0.428). Equally, contrasting NF_SMR_ and MPH (MD = −0.17, *SE* = 0.34, 95% CI [−0.84, 0.51], *t*(82) = −0.49, *p* = .874, *d* = −0.069) and NF_TBR_ and MPH (MD = −1.20, *SE* = 0.56, 95% CI [−2.31, −0.09], *t*(82) = −2.15, *p* = .087, *d* = −0.497) did not reveal significant effects.

Regarding the change in ED, a marginally non‐significant decrease was observed for NF_SMR_ (MD = −1.08, *SE* = 0.55, 95% CI [−2.18, 0.02], *t*(81) = −1.95, *p* = .054, *d* = −0.217) and NF_TBR_ (MD = −2.29, *SE* = 1.18, 95% CI [−4.65, 0.06], *t*(81) = −1.94, *p* = .056, *d* = −0.696) and a significant decrease for MPH (MD = −3.46, *SE* = 0.56, 95% CI [−4.57, −2.35], *t*(81) = −6.20, *p* = <.001, *d* = −0.142). Solely, NF_SMR_ and MPH (MD = 2.38, *SE* = 0.80, 95% CI [0.77, 3.98], *t*(81) = 2.97, *p* = .011, *d* = 0.479) differed significantly in favor of MPH, whereas no significant differences were found comparing NF_SMR_ and NF_TBR_ (MD = 1.21, *SE* = 1.33, 95% CI [−1.44, 3.87], *t*(81) = 0.91, *p* = .636, *d* = 0.244) and comparing NF_TBR_ and MPH (MD = 1.17, *SE* = 1.30, 95% CI [−1.41, 3.75], *t*(81) = 0.90, *p* = .642, *d* = 0.235).

Non‐inferiority of NF_SMR_ compared to MPH was demonstrated for both iEP outcomes (parent‐ and teacher‐ratings), but not for ED outcomes. Showing non‐inferiority of NF_TBR_ to MPH and to NF_SMR_ failed in most domains, except for the comparison with MPH iEP‐teacher‐ratings; NF_SMR_ was non‐inferior to NF_TBR_ in both parent‐rated outcomes (iEP and ED). For details, see Appendix S5.

### Predictive effects of iEP/ED on the decrease in ADHD symptoms D0–D90

Across all models, neither iEP nor ED at baseline (D0) showed significant main effects on the change in ADHD symptoms from D0 to D90, regardless of the rater source (clinician, parent, or teacher) and independent of the intervention group (NF_TBR_, NF_SMR_, MPH). Similarly, interaction effects between iEP or ED and the respective intervention groups were not significant in any of the models (all *p* > .05; see Appendices S6 and S7).

By contrast, baseline ADHD symptoms (D0) consistently and significantly predicted the change in ADHD symptoms across all analyses (iEP: clinician‐rated: *β* = −.50, *SE* = 0.09, *t* = −5.61, *p* < .001, *d* = −0.059; parent‐rated: *β* = −.48, *SE* = 0.09, *t* = −5.66, *p* < .001, *d* = −0.047; teacher‐rated: *β* = −.42, *SE* = 0.08, *t* = −5.13, *p* < .001, *d* = −0.035; ED: clinician‐rated: *β* = −.53, *SE* = 0.10, *t* = −5.29, *p* < .001, *d* = −0.062; parent‐rated: *β* = −.47, *SE* = 0.10, *t* = −4.71, *p* < .001, *d* = 0.047; teacher‐rated: *β* = −.53, *SE* = 0.12, *t* = −4.59, *p* < .001, *d* = −0.045), indicating that higher initial symptom severity predicted greater symptom reduction over time. This pattern likely reflects regression‐to‐the‐mean effects and the strong explanatory value of baseline symptom levels (see Figure 2A–2C).

**Figure 2:**
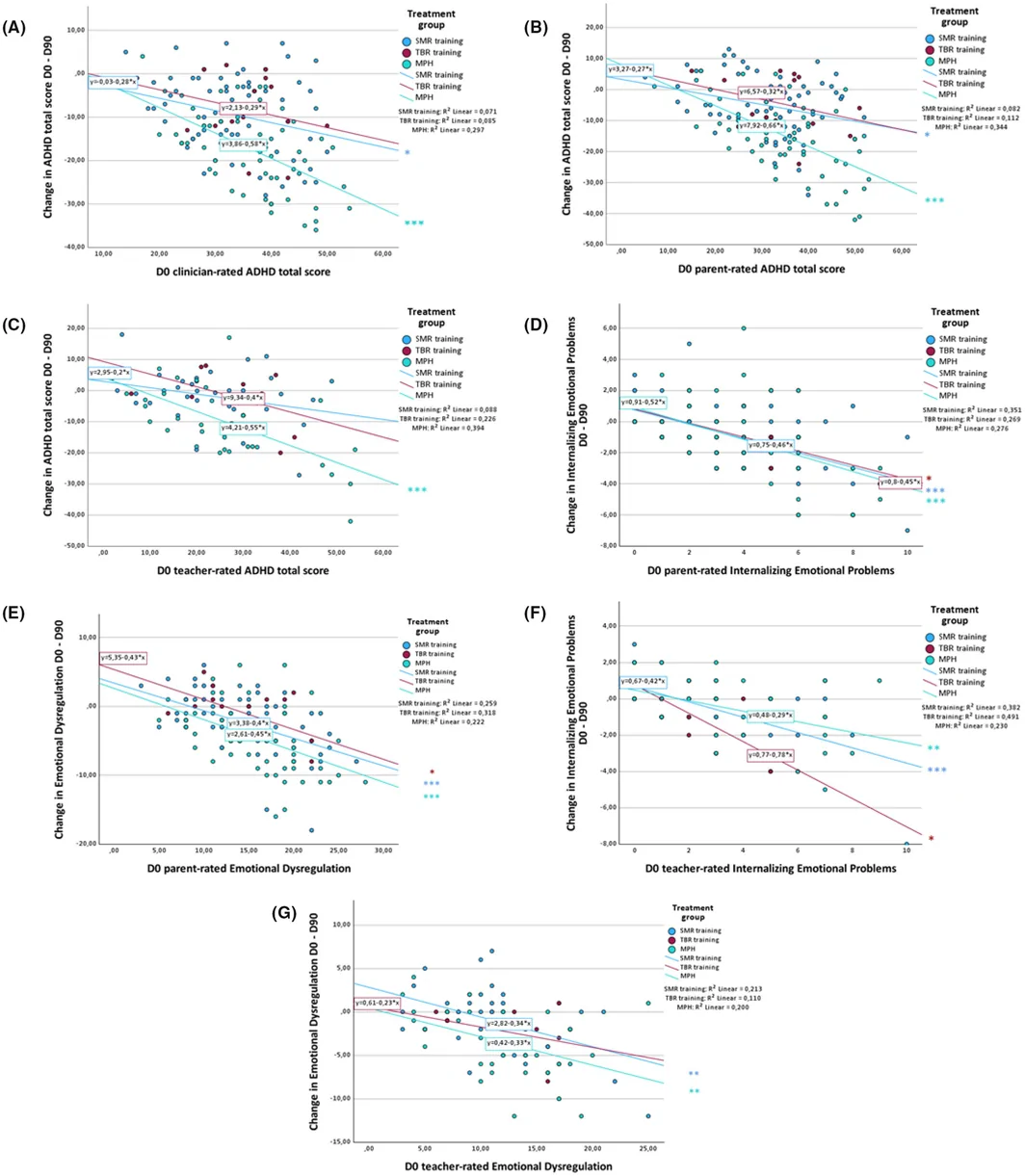
Association between ADHD and emotional scores at D0 and changes in the respective scores D0–D90. *p < .05, **p < .01, ***p < .001

Regarding covariates, age and study center occasionally showed significant or marginally non‐significant effects, whereas gender and IQ were not associated with changes in ADHD symptoms (all *p* > .05; see Appendices S6 and S7).

After applying a Bonferroni correction (*α*_adj_ = .005), only the predictive effects of baseline ADHD symptoms remained significant, as well as a specific effect within the MPH group for parent‐ and teacher‐rated outcomes in the iEP model. In a sensitivity analysis excluding baseline ADHD symptoms (D0), results were consistent with the main analyses, confirming that neither iEP nor ED significantly predicted changes in ADHD symptoms (see Appendices S8 and S9).

### Correlations between iEP/ED and neuropsychological indices of EF

A significant moderate correlation was observed between parent‐rated iEP and the total score of the BRIEF (*r* = .42, 95% CI [0.27, 0.54], *p* < .001). A similar association was observed for parent‐rated ED and the total score of the BRIEF (*r* = .68, 95% CI [0.59, 0.76], *p* < .001). No further correlations between iEP/ED and neuropsychological indices of EF were found (see Appendices S10 and S11). After Bonferroni adjustment (*α*_adj_ = .002), the correlation between both parent‐rated iEP and ED and the BRIEF remained significant.

### Predictive effects of neuropsychological indices of EF on the change in iEP/ED D0–D90

None of the neuropsychological indices showed a predictive value both in the main effect and in the interaction with the intervention groups predicting the decrease in parent‐rated iEP between D0 and D90 (Appendix S12). By contrast, iEP at D0 significantly predicted the change in iEP between D0 and D90 (*β* = −.51, *SE* = 0.08, *t* = −6.80, *p* < .001, *d* = −0.201). As iEP at D0 explained a large proportion of variance in the symptom change from D0 to D90 in all intervention groups, this was expected (see Figure 2D; see Appendix S12).

Referring to the predictive effects of neuropsychological indices of EF on the change in ED, none of them revealed a significant predictive value. However, ED at D0 significantly predicted the decrease in ED between D0 and D90 (*β* = −.48, *SE* = 0.09, *t* = −5.21, *p* < .001, *d* = −0.108; see Figure 2E; see Appendix S13).

Considering the teacher‐ratings, again none of the neuropsychological indices showed a predictive value, both in the main effect and in the interaction with the intervention groups predicting the decrease in iEP between D0 and D90. Baseline iEP significantly predicted the change in iEP between D0 and D90 (*β* = −.28, *SE* = 0.07, *t* = −3.95, *p* < .001, *d* = −0.114; see Figure 2F; see Appendix S12).

Referring to the predictive value of neuropsychological indices on the change in ED, none of the neuropsychological indices showed a predictive value, both in the main effect and in the interaction with the intervention groups predicting the decrease in ED between D0 and D90 but again, ED at D0 significantly predicted the change in ED between D0 and D90 (*β* = −.32, *SE* = 0.08, *t* = −4.00, *p* < .001, *d* = −0.065; see Figure 2G; see Appendix S13).

After a Bonferroni adjustment (*α*_adj_ = .002), the predictive value of iEP and ED at D0 remained significant, both in the parent‐ and teacher‐ratings. For the parent‐ratings, a post‐hoc sensitivity analysis, omitting iEP at D0, confirmed that none of the neuropsychological indices had a significant predictive value (see Appendix S14). By contrast, omitting ED at D0, BRIEF revealed a marginally non‐significant effect predicting the decrease in ED (*β* = −.03, *SE* = 0.01, *t* = −2.94, *p* = .055, *d* = −0.006).

Regarding the teacher‐ratings, hit reaction time and its interaction with MPH (*β* = .01, *SE* = 0.01, *t* = 1.92, *p* = .059, *d* = 0.004) revealed a marginally non‐significant predictive value, whereas omission errors (*β* = −.22, *SE* = 0.09, *t* = −2.49, *p* = .016, *d* = −0.089) and their interaction with MPH (*β* = .26, *SE* = 0.10, *t* = 2.54, *p* = .014, *d* = 0.107) revealed a significant predictive value regarding a decrease in iEP. Apart from study center, no effects were found for any other covariates (see Appendix S14). For the decrease in ED, sensitivity analysis showed a marginal significant predictive value for the interaction between omission errors and MPH (*β* = .46, *SE* = 0.24, *t* = 1.95, *p* = .056, *d* = 0.092). None of the covariates revealed a significant effect predicting the decrease in ED (see Appendix S15).

## Discussion

The aim of this study was to examine the effects of NF@Home and MPH on iEP/ED in a sample of children and adolescents with ADHD, as well as to investigate their moderating and predictive role in relation to treatment efficacy within the respective interventions. Thereby, the associations between iEP/ED and ADHD symptom dimensions and the association with neuropsychological indices of EF were explored. The main findings of the present analysis indicate that both treatments were effective in reducing iEP/ED, as reflected in both parent and teacher ratings. Non‐inferiority of NF_SMR_ compared to MPH in reducing iEP was demonstrated across both informant groups. Regarding the reduction of ED, a significant between‐group difference was observed, favoring MPH. Small to moderate correlations between iEP and ADHD symptoms were found in the clinician‐ and parent‐ratings. Regarding ED, strong correlations with ADHD symptoms were found over all raters confirming previous findings (Beheshti et al., 2020; Seymour et al., 2014). Unexpectedly, the correlation between iEP and hyperactive–impulsive symptoms did not differ from the correlation with inattentive symptoms confirming Stern et al. (2020). Regarding ED, the correlation with hyperactive–impulsive symptoms differed significantly from the correlation with inattentive symptoms in line with previous findings (Groves et al., 2020; Masi et al., 2020; Seymour et al., 2014), with a stronger association between ED and hyperactive–impulsive symptoms; this may reflect shared deficits in bottom‐up regulatory processes, whereas inattentive symptoms have been more consistently linked to impairments in top‐down control mechanisms (Martel, Nigg, & von Eye, 2009; Sonuga‐Barke, 2005). Although the evidence regarding iEP remains inconclusive, it is plausible that both bottom‐up and top‐down processes contribute to iEP, given findings demonstrating associations with both inattentive and hyperactive–impulsive symptom domains (Stern et al., 2020).

As the correlations between ED and ADHD were stronger over all raters compared to the correlations between iEP and ADHD, there is a higher overlap between the broader concept of ED including internalizing as well as externalizing aspects and ADHD than with internalizing aspects. This finding, however, appears unsurprising insofar as the measure used in the present study to assess ED primarily captures externalizing symptoms that align closely with the diagnostic criteria for ADHD. Regarding the initial debate on considering ED a further cardinal symptom of ADHD, our results are more in line with a correlated but still distinct construct; however, this also depends substantially on the definition applied, the underlying conceptual framework, and the specific operationalization of the construct. The results further highlight that internalizing aspects also play a distinct role within the context of the externalizing symptomatology of ADHD.

Regarding the effectiveness of NF@Home and MPH decreasing iEP and ED, taking into account interrater discrepancies, the results indicate a comparable efficacy of both treatments for additional emotion‐specific processes, especially internalizing symptoms. However, for both groups, effects could also be interpreted as unspecific or represent spontaneous remissions.

Interrater discrepancies were more pronounced between parent‐ and teacher‐ratings for iEP than for ED, although not significantly different, possibly due to ED comprising multiple SDQ subscales (De Los Reyes et al., 2015). Teachers may be more attuned to externalizing symptoms, while internalizing symptoms—especially in boys—may be masked in classroom settings (Bussalb et al., 2019; Fernández De La Cruz et al., 2015). Lower baseline teacher‐ratings and a high proportion of missing data may further account for weaker associations and treatment effects in teacher‐reported outcomes. These findings underscore the importance of addressing emotion‐specific processes in ADHD and caution against relying solely on a single informant.

In contrast to previous evidence (Galera et al., 2021; Masi et al., 2020), both iEP and ED did not reveal a predictive value for the change in ADHD‐symptoms. Performing a sensitivity analysis, omitting baseline ADHD symptoms, these results remained stable. Contrary to this, Masi et al. (2020) observed a predictive value of ED for positive changes in ADHD symptoms with MPH. As ED proved to be a rather correlated but still distinct construct, the recent results did not indicate ED being a further cardinal symptom of ADHD. This conclusion is drawn by the expectation that a cardinal symptom would explain sufficient variance in symptomatology having a predictive value for its change. This can be supported by the finding that ED revealed no predictive value in the post‐hoc sensitivity analysis, despite the omission of baseline ADHD symptoms.

In line with previous findings (Biederman et al., 2020), moderate/strong correlations were found between iEP/ED and the BRIEF. Other neuropsychological indices of EF using the CPT did not show significant correlations. This may be due to the BRIEF being a subjective measure—as are the ratings of ED and iEP—whereas the neuropsychological indices of CPT are typically interpreted as “objective” measures, probably measuring distinct aspects and additional facets (Poh, Lim, Chew, Lim‐Ashworth, & Lim, 2024).

Regarding the change in iEP and ED, baseline severity proved to be the strongest predictor. Neuropsychological indices did not show a predictive value for its change, confirming Wåhlstedt, Thorell, and Bohlin (2008). There were studies that found a predictive value of neuropsychological indices, but this was completely annulled, including ADHD symptoms as a control variable (Banaschewski et al., 2012; Groves et al., 2022). In both prediction models, the strongest predictive value for a symptom change was observed for baseline symptoms, suggesting that those explain a large proportion of variance in symptom change. Due to multiple testing, multicollinearity is suspected at this point, limiting the predictive value of individual predictors (Zhang & Castelló, 2017).

### Theoretical and clinical implications

From a theoretical perspective, the present study highlights the importance of emotional problems in the context of ADHD and thus makes a valuable contribution to further characterizing this heterogeneous phenotype and associated concepts. Thus, our findings clearly emphasize the need to describe symptoms at the individual level and to address them in an adaptive treatment, while also considering comorbid and transdiagnostically‐relevant concepts.

Considering the correlations between iEP/ED and ADHD symptoms, broad diagnostic characterization and developing strategies to improve or overcome emotional problems could be promising for patients with ADHD. Studies have shown that Dialectical Behavioral Therapy (DBT), primarily developed for treating borderline personality disorder, was effective in treating ED within an adolescent/adult‐ADHD sample (Easdale‐Cheele et al., 2024; Hesslinger et al., 2002; Meyer, Zetterqvist, Unenge Hallerbäck, Ramklint, & Isaksson, 2022). Especially, teaching emotion‐regulation strategies was found to have a major effect (Meyer et al., 2022).

Regarding the relevance of neuropsychological indices of EF in clinical practice, the correlation observed between neuropsychological deficits reported within the BRIEF and iEP/ED suggests that training of EF may be promising in improving iEP/ED—although objective measures do not support this conclusion. In line with this, Schweizer, Grahn, Hampshire, Mobbs, and Dalgleish (2013) found that the training of emotional working memory improved emotion regulation, probably associated with an increased activity in PFC, which is closely linked to EF (Margari et al., 2016; Shimamura, 2000).

### Limitations

Several limitations have to be mentioned within the context of the current work. A shortcoming is the missing clinician‐rating of iEP/ED. As clinician‐ratings are mainly based on parent‐ratings, especially for younger children, parent‐ratings were used for analyses. Due to interrater discrepancies (De Los Reyes et al., 2015), the reliability of the results would probably be higher if the assessment had been made by the same rater. Due to an intercorrelation between parent‐ and teacher‐ratings, not both were included as predictors for clinician‐rating, avoiding an increased likelihood of multicollinearity and therefore the risk of an unstable prediction.

Considering design‐based limitations, the lack of a non‐active control group should be mentioned. The natural course could not be observed, and therefore, the extent of the effects being due to intervention or natural course is uncertain. However, the omission of a non‐active control group can be justified for ethical reasons. Additionally, there was no sham‐NF. However, concerning ethical aspects of false feedback (Holtmann, Pniewski, Wachtlin, Wörz, & Strehl, 2014) and the risk of becoming aware of false feedback, missing sham‐NF seems to be tolerable.

A further limitation was the impossibility of blinding the patients, parents, and clinicians, possibly leading to expectation effects (Gevensleben et al., 2014).

Finally, the lack of follow‐up observations should be mentioned. These are relevant, particularly for NF, as its effects often appear later (Purper‐Ouakil et al., 2022; Van Doren et al., 2019).

## Conclusions

The aim of this study was to examine the treatment effects of individualized NF@Home and MPH on iEP/ED in children and adolescents with ADHD, as well as to investigate their moderating and predictive role in relation to treatment efficacy. Recent findings provide relevant insights into the associations between iEP/ED and ADHD symptoms, confirming their co‐occurrence and emphasizing the need to distinguish between externalizing and internalizing aspects of emotional problems in diagnostic assessment. The results underline the heterogeneity of the ADHD phenotype. In line with the ongoing debate, ED appears to be a correlated but distinct construct frequently observed in ADHD. However, this conclusion also depends substantially on the definition applied, the underlying conceptual framework, and the specific operationalization of the construct, with conceptual ambiguities further complicating a clear methodological delineation.

The findings indicate no superiority of MPH over NF@Home in treating iEP, but a superiority of MPH in treating ED. Given the limited consensus on effective treatments for iEP/ED in ADHD, both interventions might be beneficial, though effects may differ. Due to the lack of a control group, results should be interpreted cautiously. Nevertheless, they highlight the need for individualized, possibly multimodal treatment approaches combining pharmacological and non‐pharmacological methods. Furthermore, the development and implementation of interventions targeting emotional problems and emotion regulation may represent a promising avenue for clinical practice in individuals with ADHD.

Further research is required on the moderating role of iEP/ED in ADHD treatment. While baseline ADHD symptoms were the strongest predictor, iEP/ED showed limited predictive value for changes in ADHD symptoms under MPH and NF@Home. As age significantly covaried with treatment outcomes, studying older samples—particularly during developmental transitions—may yield additional insights.

Finally, neuropsychological indices of EF showed limited predictive value for changes in iEP/ED. Given scarce evidence, future research should clarify their clinical relevance for individualized treatment planning.

## Ethical considerations

Informed consent (and assent where applicable) was obtained from all participants and their legal guardians prior to participation. Ethical approval was granted by all local ethic committees prior to the study start (2016/08) at different dates for the different sites. For Belgium: Comité d'Ethique hospital facultaire ERASME‐ULB Bruxelles (07/11/2016), for France: Comité de Protection des Personnes Sud‐Méditerranée I (20/04.2016), for Germany: Medizinische Ethikkommission II der Medizinischen Fakulät Mannheim (23/09/2016), for Spain: Dictamen del Comite de etica de la investigacion con medicamentos‐Madrid (26/07/2016), and for Switzerland: Kantonale Ethikkommission Zürich (23/03/2017).

Approval/reference numbers: ID‐RCB: 2016‐A00339‐4; EudraCT: 2016–002171‐94; Mannheim ethic commission no.: 2016‐404MF‐MA.

## Trial registration

The trial was registered with the US National Institute of Health at ClinicalTrials.gov (Identifier: NCT02778360). The protocol was first submitted on 12 May 2016 and is available online at https://clinicaltrials.gov/study/NCT02778360.

**Key points**

**What's known?**

- Emotional dysregulation (ED) and emotional problems (EP) are common in ADHD: Previous studies have identified a strong association between ED and ADHD, particularly with hyperactive–impulsive symptoms. This study confirms these associations and emphasizes that ED, including both internalizing and externalizing problems, frequently co‐occurs with ADHD.

**What's new?**

- Comparable effects of NF@Home and MPH on internalizing emotional problems (iEP): Both neurofeedback at home (NF@Home) and methylphenidate (MPH) were found to be probably effective in reducing iEP, with no significant difference in effectiveness across interventions. However, due to the lack of a control group, unspecific effects or effects due to the natural course (of development) cannot be ruled out.
- Limited predictive value of emotional symptoms and executive function (EF): Contrary to some previous findings, neither iEP nor ED predicted changes in ADHD core symptoms under treatment. Similarly, neuropsychological measures of EF, especially objective ones, did not predict improvements in iEP/ED—highlighting the complexity and multifactorial nature of ADHD treatment response.

**What's relevant?**

- Personalized treatment needed: The findings underline ADHD's clinical heterogeneity and call for individualized treatment plans that consider comorbid, transdiagnostically relevant symptoms.
- Personalized diagnosis and importance of multi‐informant assessments: Differences between parent and teacher ratings emphasize the need for clinicians to integrate multiple perspectives. Emotional symptoms, especially internalizing ones, may be underestimated in school settings, reinforcing the importance of broad, context‐sensitive diagnostics.

## Supporting information


**Appendix S1.** Exclusion criteria.
**Appendix S2.** Sociodemographic data, clinical, emotional, and neuropsychological characteristics of PP population at baseline (D0).
**Appendix S3.** Correlations between ADHD symptoms and SDQ‐emotional problems at D0.
**Appendix S4.** Correlations between ADHD symptoms and SDQ‐emotional dysregulation at D0.
**Appendix S5.** Non‐inferiority results for iEP and ED, evaluated according to the two margin approaches.
**Appendix S6.** Regression analysis of between‐group effects predicting the treatment response based on the severity of SDQ‐emotional problems at D0.
**Appendix S7.** Regression analysis of between‐group effects predicting the treatment response based on the severity of SDQ‐emotional dysregulation at D0.
**Appendix S8.** Sensitivity analysis of regression analysis of between‐group effects predicting the treatment response based on the severity of SDQ‐emotional problems at D0.
**Appendix S9.** Sensitivity analysis of regression analysis of between‐group effects predicting the treatment response based on the severity of SDQ‐emotional dysregulation at D0.
**Appendix S10.** Correlations between neuropsychological indices and SDQ‐emotional problems at D0.
**Appendix S11.** Correlations between neuropsychological indices and SDQ‐emotional dysregulation at D0.
**Appendix S12.** Regression analysis of between‐group effects predicting the change in SDQ‐emotional problems between D0 and D90 based on neuropsychological indices at D0.
**Appendix S13.** Regression analysis of between‐group effects predicting the change in SDQ‐emotional dysregulation between D0 and D90 based on neuropsychological indices at D0.
**Appendix S14.** Sensitivity analysis of regression analysis of between‐group effects predicting the change in SDQ‐emotional problems between D0 and D90 based on neuropsychological indices at D0.
**Appendix S15.** Sensitivity analysis of regression analysis of between‐group effects predicting the change in SDQ‐emotional dysregulation between D0 and D90 based on neuropsychological indices at D0.

## Data Availability

Research data are not shared. The data cannot be made available due to privacy or ethical restrictions.
