# Supplementary material for: Emotional problems and dysregulation in children/youth with ADHD: predictive and moderating effects in a randomized controlled trial of methylphenidate and neurofeedback
Source: J Child Psychol Psychiatry. 2026 Jul 28;67(12):2069–83. doi: 10.1111/jcpp.70205 (PMC13745781; doi:10.1111/jcpp.70205)
Supplement: Supplementary file 1 [file JCPP-67-2069-s001.docx]

**Emotional Problems and Dysregulation in Children/Youth with ADHD: Predictive and Moderating Effects in a Randomized Controlled Trial of Methylphenidate and Neurofeedback**

**Supporting Information**

**Appendix S1.**

*Exclusion criteria*

Study exclusion was intended if (1) a patient was diagnosed with the predominantly hyperactive-impulsive subtype, (2) comorbid psychiatric or neurological disorders (epilepsy, autism, schizophrenia, generalized anxiety disorder, major depression, tic disorder) were diagnosed, (3) psychoactive or NF treatment already took place or (4) the medication of a physical disease interfered with psychoactive substances. Furthermore, (5) patients with a chronic physical disorder were excluded, (6) another treatment was planned within the following six months and (7) the IQ was < 80. Additionally, patients were excluded if (8) there was a risk of suicide attempt, (9) the family had relocation plans and (10) the appropriate NF application was not ensured (Bioulac et al., 2019).

**Appendix S2.**

*Sociodemographic data, clinical, emotional and neuropsychological characteristics of PP population at baseline (D0)*

| Variable | SMR^a^-training | TBR^b^-training | MPH^c^-group | Group comparison |
| --- | --- | --- | --- | --- |
|  | *N*(%) *M(SD)* Min Max | *N*(%) *M(SD)* Min Max | *N*(%) *M(SD)* Min Max | Test estimates *p* |
| Gender | 72(100) | 15(100) | 59(100) | 1.42 (χ^2^) .491 |
| Male | 55(76.4) | 12(80.0) | 50(84.7) |  |
| Female | 17(23.6) | 3(20.0) | 9(15.3) |  |
| Age | 10.08(1.89) 7 13 | 9.00(1.07) 7 10 | 9.37(1.77) 7 13 | 2.00 (*F*) .141 |
| IQ | 109.61(15.90) 80 147 | 111.40(18.42) 94 154 | 105.39(14.88) 80 136 | 1.24 (*F*) .293 |
| Clinician |  |  |  |  |
| ADHD-RS-total^d^ | 33.75(8.51) 14 50 | 35.07(8.29) 16 50 | 36.27(8.57) 17 54 | 1.32 (*F*) .273 |
| ADHD-RS-IA^e^ | 19.83(3.88) 9 27 | 19.20(4.07) 11 27 | 9.85(3.03) 10 27 | .41 (*F*) .667 |
| ADHD-RS-HI^f^ | 13.86(6.88) 0 25 | 15.87(5.68) 2 23 | 15.98(6.61) 4 27 | 1.39 (*F*) .255 |
| Parents |  |  |  |  |
| ADHD-RS-total | 31.25(9.87) 5 52 | 34.19(9.43) 15 51 | 35.27(10.56) 7 53 | 1.81 (*F*) .169 |
| ADHD-RS-IA | 18.11(5.43) 4 27 | 17.85(3.37) 13 25 | 19.45(5.53) 6 27 | .41 (*F*) .667 |
| ADHD-RS-HI | 12.85(6.47) 0 26 | 16.29(6.49) 2 26 | 15.64(7.24) 1 27 | 2.80 (*F*) .066 |
| SDQ^g^-emotional problems | 3.76(2.69) 0 10 | 3.27(2.12) 0 6 | 3.91(2.44) 0 9 | .07 (*F*) .934 |
| SDQ-emotional dysregulation^h^ | 14.47(4.50) 5 25 | 14.93(4.11) 8 22 | 15.95(4.25) 7 26 | 1.96(*F*) .146 |
| Teacher |  |  |  |  |
| ADHD-RS-total | 25.29(11.74) 2 49 | 27.20(10.08) 6 41 | 26.98(12.53) 5 54 | .03 (*F*) .966 |
| ADHD-RS-IA | 15.05(6.04) 2 27 | 13.56(6.02) 4 25 | 15.47(5.98) 2 27 | .40 (*F*) .670 |
| ADHD-RS-HI | 10.46(7.22) 0 25 | 13.64(6.36) 1 21 | 11.53(8.00) 0 27 | .58 (*F*) .560 |
| SDQ-emotional problems | 2.60(2.45) 0 10 | 2.92(1.44) 0 5 | 2.84(2.57) 0 9 | .45 (*F*) .640 |
| SDQ-emotional dysregulation | 10.86(5.20) 3 25 | 12.69(3.43) 6 17 | 12.30(5.10) 3 25 | 1.06(*F*) .349 |
| Neuropsychological indices |  |  |  |  |
| BRIEF^i^-total | 186.10(31.26) 108 251 | 193.67(26.38) 146 235 | 191.81(26.07) 122 247 | 1.04 (*F*) .358 |
| CPT^i^-omission errors | 5.52(7.44) 0.00 52.78 | 7.55(6.92) 0.00 23.26 | 9.02(12.69) 0.35 64.44 | 1.10 (*F*) .337 |
| CPT-commission errors | 52.02(19.79) 0.00 93.06 | 50.74(18.13) 20.83 79.17 | 53.56(20.39) 2.78 84.72 | .10 (*F*) .903 |
| CPT-hit reaction time (ms) | 471.90(103.25) 296.47 873.72 | 509.42(101.40) 371.00 655.60 | 503.37(101.75) 368.66 849.92 | .41 (*F*) .665 |
| CPT-variability of hit reaction time  (ms) | 99.14(77.11) 437.81 22.34 | 109.22(69.68) 9.01 226.98 | 114.65(69.04) 324.21 16.25 | .31 (*F*) .735 |

*Notes.* ^a^Sensorimotor rhythm. ^b^Theta-Beta-ratio. ^c^Methylphenidate. ^d^Represents the total score of the inattentive and hyperactive-impulsive sub scores. ^e^Inattentive sub score. ^f^Hyperactive-impulsive sub score. ^g^Strenghts and Difficulties Questionnaire. ^h^Represents the sum score of the SDQ sub scales emotional problems, conduct problems and hyperactivity. ^i^Behavior Rating Inventory Executive Function. ^i^Conners Continuous Performance Test.

**Appendix S3.**

*Correlations between ADHD symptoms and SDQ-emotional problems at D0*

| Variable | ADHD-RS^a^-C^b^-total | ADHD-RS-C-IA^c^ | ADHD-RS-C-HI^d^ | | ADHD-RS-P^e^-total | | ADHD-RS-P-IA | | ADHD-RS-P-HI | | ADHD-RS-T^f^-total | | ADHD-RS-T-IA | | ADHD-RS-T-HI | | SDQ^g^-P-emotional problems | | SDQ-T-emotional problems | |  |
| --- | --- | --- | --- | --- | --- | --- | --- | --- | --- | --- | --- | --- | --- | --- | --- | --- | --- | --- | --- | --- | --- |
|  | *r[CI]* ^h^  *p*^i^ | *r[CI]*  *p* | *r[CI]*  *p* | | *r[CI]*  *p* | | *r[CI]*  *p* | | *r[CI]*  *p* | | *r[CI]*  *p* | | *r[CI]*  *p* | | *r[CI]*  *p* | | *r[CI]*  *p* | | *r[CI]*  *p* | |  |
| ADHD-RS-C-total | 1 |  |  | |  | |  | |  | |  | |  | |  | |  | |  | |  |
| ADHD-RS-C-IA | **.65[.55; .74]**  **< .001** | 1 |  | |  | |  | |  | |  | |  | |  | |  | |  | |  |
| ADHD-RS-C-HI | **.89[.85; .92]**  **< .001** | **.23[.07; .38]**  **.004** | | 1 | |  | |  | |  | |  | |  | |  | |  | |  | |
| ADHD-RS-P-total | **.73[.64; .79]**  **< .001** | .**54[.42; .65]**  **< .001** | **.61[.49; .70]**  **< .001** | | 1 | |  | |  | |  | |  | |  | |  | |  | |  |
| ADHD-RS-P-IA | **.39[.24; .52]**  **< .001** | **.61[.50; .71]**  **< .001** | .14[-.03; .30]  .100 | | **.69[.59; .77]**  **< .001** | | 1 | |  | |  | |  | |  | |  | |  | |  |
| ADHD-RS-P-HI | **.65[.54; .73]**  **< .001** | **.23[.07; .38]**  **.005** | **.68[.58; .76]**  **< .001** | | **.80[.73; .85]**  **< .001** | | **.40[.26; .53]**  **< .001** | | 1 | |  | |  | |  | |  | |  | |  |
| ADHD-RS-T-total | **.26[.09; .42]**  **.004** | .04[-.14; .22]  .633 | **.31[.14; .47]**  **< .001** | | .15[-.03; 32]  .098 | | -.03[-.21; .17]  .768 | | **.20[.02; .37]**  **.032** | | 1 | |  | |  | |  | |  | |  |
| ADHD-RS-T-IA | .16[-.02; .33]  .084 | .18[-.01; .34]  .056 | .09[-.09; .27]  .306 | | .04[-.15; .21]  .708 | | .04[-.15; .22]  .707 | | -.02[-.20; .16]  .813 | | **.85[.79; .89]**  **< .001** | | 1 | |  | |  | |  | |  |
| ADHD-RS-T-HI | **.29[.12; .45]**  **.001** | -.07[-.25; .11]  .426 | **.43[.27; .57]**  **< .001** | | **.23[.05; .39]**  **.013** | | -.07[-.15; .22]  .477 | | .35[.18; .50]  < .001 | | **.90[.86; .93]**  **< .001** | | **.54[.40; .67]**  **< .001** | | 1 | |  | |  | |  |
| SDQ-P-emotional problems | **.22[.06; .37]**  **.008** | **.241[.08; .39]**  **.003** | .14[-.03; .29]  .098 | | **.26[.11; .41]**  **.001** | | **.18[.01; .33]**  **.038** | | **.20[.04; .36]**  **.015** | | **-.22[-.38;**  **-.04]**  **.018** | | **-.22[-.39;**  **-.04]**  **.015** | | -.14[-.31; .04]  .130 | | 1 | |  | |  |
| SDQ-T-emotional problems | .10[-.08; .27]  .275 | .032[-.15; .21]  .730 | .11[-.07; .28]  .228 | | .05[-. 14; .22]  .622 | | -.11[-.29; .07]  .225 | | -.01[-.19; .18]  .940 | | .08[-.10; .26]  .378 | | .15[-.04; .32]  .116 | | .03[-.15; .21]  .744 | | **.23[.05; .39]**  **.013** | | 1 | |  |

*Notes.* ^a^Represents the total score of the inattentive and hyperactive-impulsive sub scores. ^b^Clinician-rating. ^c^Inattentive sub score. ^d^Hyperactive-impulsive sub score. ^e^Parent-rating. ^f^Teacher-rating. ^g^Strenghts and Difficulties Questionnaire. ^h^95% Confidence interval (two-sided). Estimates are based on Fisher´s r/z transformation. ^i^*p*-values less than .05 are highlighted in bold.

**Appendix S4.**

*Correlations between ADHD symptoms and SDQ-emotional dysregulation at D0*

| Variable | ADHD-RS^a^-C^b^-total | ADHD-RS-C-IA^c^ | ADHD-RS-C-HI^d^ | | ADHD-RS-P^e^-total | | ADHD-RS-P-IA | | ADHD-RS-P-HI | | ADHD-RS-T^f^-total | | ADHD-RS-T-IA | | ADHD-RS-T-HI | | SDQ-P-emotional dysregulation^g^ | | SDQ-T-emotional dysregulation | |
| --- | --- | --- | --- | --- | --- | --- | --- | --- | --- | --- | --- | --- | --- | --- | --- | --- | --- | --- | --- | --- |
|  | *r[CI]* ^h^  *p*^i^ | *r[CI]*  *p* | *r[CI]*  *p* | | *r[CI]*  *p* | | *r[CI]*  *p* | | *r[CI]*  *p* | | *r[CI]*  *p* | | *r[CI]*  *p* | | *r[CI]*  *p* | | *r[CI]*  *p* | | *r[CI]*  *p* | |
| ADHD-RS-C-total | 1 |  |  | |  | |  | |  | |  | |  | |  | |  | |  | |
| ADHD-RS-C-IA | **.65[.55; .74]**  **< .001** | 1 |  | |  | |  | |  | |  | |  | |  | |  | |  | |
| ADHD-RS-C-HI | **.89[.85; .92]**  **< .001** | **.23[.07; .38]**  **.004** | | 1 | |  | |  | |  | |  | |  | |  | |  | |  |
| ADHD-RS-P-total | **.73[.64; .79]**  **< .001** | .**54[.42; .65]**  **< .001** | **.61[.49; .70]**  **< .001** | | 1 | |  | |  | |  | |  | |  | |  | |  | |
| ADHD-RS-P-IA | **.39[.24; .52]**  **< .001** | **.61[.50; .71]**  **< .001** | .14[-.03; .30]  .100 | | **.69[.59; .77]**  **< .001** | | 1 | |  | |  | |  | |  | |  | |  | |
| ADHD-RS-P-HI | **.65[.54; .73]**  **< .001** | **.23[.07; .38]**  **.005** | **.68[.58; .76]**  **< .001** | | **.80[.73; .85]**  **< .001** | | **.40[.26; .53]**  **< .001** | | 1 | |  | |  | |  | |  | |  | |
| ADHD-RS-T-total | **.26[.09; .42]**  **.004** | .04[-.14; .22]  .633 | **.31[.14; .47]**  **< .001** | | .15[-.03; 32]  .098 | | -.03[-.21; .17]  .768 | | **.20[.02; .37]**  **.032** | | 1 | |  | |  | |  | |  | |
| ADHD-RS-T-IA | .16[-.02; .33]  .084 | .18[-.01; .34]  .056 | .09[-.09; .27]  .306 | | .04[-.15; .21]  .708 | | .04[-.15; .22]  .707 | | -.02[-.20; .16]  .813 | | **.85[.79; .89]**  **< .001** | | 1 | |  | |  | |  | |
| ADHD-RS-T-HI | **.29[.12; .45]**  **.001** | -.07[-.25; .11]  .426 | **.43[.27; .57]**  **< .001** | | **.23[.05; .39]**  **.013** | | -.07[-.15; .22]  .477 | | .35[.18; .50]  < .001 | | **.90[.86; .93]**  **< .001** | | **.54[.40; .67]**  **< .001** | | 1 | |  | |  | |
| SDQ-P-emotional dysregulation | **.53[.40; .64]**  **<.001** | **.26[.11; .41]**  **.001** | **.53[.40; .63]**  **<.001** | | **.60[.49; .70]**  **<.001** | | **.24[.08; .39]**  **.004** | | **.57[.48; .69]**  **<.001** | | .05[-.13;  .23]  .600 | | -.11[-.29;  -.07]  .240 | | **.18[.00; .35]**  **.050** | | 1 | |  | |
| SDQ-T-emotional dysregulation | **.30[.13; .46]**  **<.001** | .07[-.11; .25]  .421 | **.35[.18; .50]**  **<.001** | | .18[-.001; .35]  .052 | | -.06[-.24; .13]  .536 | | .16[-.02; .34]  .077 | | **.73[.63; .80]**  **<.001** | | **.64[.52; .74]**  **<.001** | | **.64[.52; .74]**  **<.001** | | .16[-.02;  .33]  .079 | | 1 | |

*Notes.* ^a^Represents the total score of the inattentive and hyperactive-impulsive sub scores. ^b^Clinician-rating. ^c^Inattentive sub score. ^d^Hyperactive-impulsive sub score. ^e^Parent-rating. ^f^Teacher-rating. ^g^Represents the sum score of the SDQ sub scales emotional problems, conduct problems and hyperactivity. ^h^95% Confidence interval (two-sided). Estimates are based on Fisher´s r/z transformation. ^i^*p*-values less than .05 are highlighted in bold.

**Appendix S5.**

*Non-Inferiority results for iEP and ED, evaluated according to the two margin approaches*

| **Comparison** | Variable | Mean difference DD^a^ | Margin (Literature^b^,  Δ=0.2\Delta = 0.2) | NI^c^ (Literature) | Margin^d^ (0.3 × SD) | NI (0.3×SD) |
| --- | --- | --- | --- | --- | --- | --- |
| **NF_SMR_ vs MPH** | iEP (parent) | 0.10 | 0.20 | Yes | 0.234 | Yes |
|  | iEP (teacher) | -0.16 | 0.20 | Yes | 0.261 | Yes |
|  | ED (parent) | 2.28 | 0.20 | No | 0.36 | No |
|  | ED (teacher) | 2.56 | 0.20 | No | 0.69 | No |
| **NF_TBR_ vs MPH** | iEP (parent) | 0.44 | 0.20 | No | 0.234 | No |
|  | iEP (teacher) | -1.33 | 0.20 | Yes | 0.261 | Yes |
|  | ED (parent) | 3.72 | 0.20 | No | 0.36 | No |
|  | ED (teacher) | 1.09 | 0.20 | No | 0.69 | No |
| **NF_SMR_ vs NF_TBR_** | iEP (parent) | -0.33 | 0.20 | Yes | 0.234 | Yes |
|  | iEP (teacher) | 1.18 | 0.20 | No | 0.261 | No |
|  | ED (parent) | -1.44 | 0.20 | Yes | 0.36 | Yes |
|  | ED (teacher) | 1.47 | 0.20 | No | 0.69 | No |

*Notes.* ^a^mean difference of change scores (positive = reference group stronger, negative = test group stronger. ^b^fixed at 0.2 (conservative). ^c^non-inferior (Yes = D≤ΔD \leq \Delta, N0 = D>ΔD > \Delta). ^d^margin based on 0.3 × SD of the change scores (less conservative).

**Appendix S6.**

*Regression analysis of between-group effects predicting the treatment response based on the severity of SDQ-emotional problems at D0*

| Variable | Clinician | Parents | Teacher |
| --- | --- | --- | --- |
|  | β SE t *p*^a^ *d* | β SE t *p d* | β SE t *p d* |
| SDQ^b^-emotional problems D0 | -.33 0.37 -.89 .377 -.039 | -.33 0.43 -0.75 .455 -.032 | -.29 0.56 -0.52 .603 -.025 |
| SMR (dummy-coded, reference category) |  |  |  |
| TBR^c^ | 2.66 4.38 .61 .544 .312 | 2.80 5.06 0.55 .581 .278 | -5.92 7.09 -0.83 .407 -.498 |
| MPH^d^ | **-8.12 2.71 -3.00 .003 -.952** | **-9.66 3.16 -3.06 .003 -.958** | **-11.20 2.86 -3.92 < .001 -.946** |
| ADHD-RS^e^-total D0 | **-.50 0.09 -5.61 < .001 -.059** | **-.48 0.09 -5.66 < .001 -.047** | **-.42 0.08 -5.13 < .001 -.035** |
| Gender | .05 1.77 .03 .979 .006 | 1.03 2.05 .50 .615 .102 | 2.79 2.53 1.10 .273 .235 |
| Study center | **-.64 0.27 -2.34 .021 -.075** | -.41 0.32 -1.29 .200 -.041 | -.17 0.37 -0.48 .635 -.015 |
| Age | **-1.11 0.42 -2.65 .009 -.130** | -.90 0.47 -1.92 .057 -.089 | -.49 0.57 -0.85 .496 -.041 |
| IQ | .06 0.05 1.30 .197 .007 | .08 0.05 1.51 .134 .008 | -.01 0.06 0.21 .837 -.001 |
| SDQ-emotional problems D0*SMR  (dummy-coded, reference category) |  |  |  |
| SDQ-emotional problems D0*TBR | -.60 1.12 -.54 .590 -.071 | -.56 1.29 -0.43 .665 -.056 | 2.03 2.03 1.00 .321 .171 |
| SDQ-emotional problems D0*MPH | .20 0.58 .34 .732 .023 | .34 0.67 0.51 .613 .034 | 1.14 0.76 1.49 .141 .096 |

*Notes.* ^a^*p*-values less than .05 are highlighted in bold. ^b^Strenghts and Difficulties Questionnaire. ^c^Theta-Beta-ratio. ^d^Methylphenidate. ^e^Represents the total score of the inattentive and hyperactive-impulsive sub scores.

**Appendix S7.**

*Regression analysis of between-group effects predicting the treatment response based on the severity of SDQ-emotional dysregulation at D0*

| Variable | Clinician | Parents | Teacher |
| --- | --- | --- | --- |
|  | β SE t *p*^a^ *d* | β SE t *p d* | β SE t *p d* |
| SDQ-emotional dysregulation^b^ D0 | .25 0.24 1.04 .300 .030 | .11 0.30 0.36 .721 .011 | .36 0.34 1.06 .294 .031 |
| SMR (dummy-coded, reference category) |  |  |  |
| TBR^c^ | 12.88 8.95 1.44 .152 1.510 | 11.78 10.44 1.13 .261 1.170 | -3.80 11.26 -0.34 .737 -.320 |
| MPH^d^ | -6.08 5.42 -1.12 .264 -.712 | -4.40 6.32 -0.70 .488 -.044 | -9.06 4.98 -1.82 .073 -.763 |
| ADHD-RS^e^-total D0 | **-.53 0.10 -5.29 < .001 -.062** | **-.47 0.10 -4.71 < .001 .047** | **-.53 0.12 -4.59 < .001 -.045** |
| Gender | .50 1.77 .28 .779 .058 | 1.22 2.06 0.59 .555 .121 | 2.51 2.57 0.98 .332 .211 |
| Study center | **-.56 0.28 -1.98 .050 -.066** | -.41 0.33 -1.24 .216 -.041 | -.11 0.36 -0.31 .754 -.009 |
| Age | **-.97 0.42 -2.30 .028 -.114** | **-.98 0.48 -2.07 .040 -.097** | -.51 0.58 -0.88 .382 -.043 |
| IQ | .05 0.05 1.06 .292 .006 | .07 0.05 1.26 .209 .007 | -.02 0.06 -0.28 .784 -.001 |
| SDQ-emotional dysregulation D0*SMR  (dummy-coded, reference category) |  |  |  |
| SDQ-emotional dysregulation D0*TBR | -.82 0.58 -1.40 .163 -.100 | -.72 0.68 -1.06 .291 -.072 | .29 0.83 0.35 .729 .024 |
| SDQ-emotional dysregulation D0*MPH | -.11 0.34 -.33 .739 -.013 | -.27 0.40 -0.67 .505 -.026 | .06 0.39 0.16 .872 .005 |

*Notes.* ^a^*p*-values less than .05 are highlighted in bold. ^b^Represents the sum score of the SDQ sub scales emotional problems, conduct problems and hyperactivity. ^c^Theta-Beta-ratio. ^d^Methylphenidate. ^e^Represents the total score of the inattentive and hyperactive-impulsive sub scores.

**Appendix S8.**

*Sensitivity analysis of regression analysis of between-group effects predicting the treatment response based on the severity of SDQ-emotional problems at D0*

| Variable | Clinician | Parents | Teacher |
| --- | --- | --- | --- |
|  | β SE t *p*^a^ *d* | β SE t *p d* | β SE t *p d* |
| SDQ^b^-emotional problems D0 | -.42 0.41 -1.03 .304 -.050 | -.64 0.48 -1.34 .183 -.064 | -.60 0.64 -.94 .352 -.051 |
| SMR (dummy-coded, reference category) |  |  |  |
| TBR^c^ | 5.65 4.82 1.17 .243 .662 | 3.60 5.63 .64 .523 .357 | -4.54 8.18 -.56 .581 -.383 |
| MPH^d^ | **-7.56 3.00 -2.52 .013 -.887** | **-10.40 3.51 -2.98 .004 -1.040** | **-11.90 3.30 -3.59 < .001 -1.000** |
| Gender | -.92 1.95 -.47 .640 -.107 | 1.11 2.28 .49 .627 .110 | .87 2.88 .30 .765 .073 |
| Study center | -.38 0.30 -1.26 .211 -.044 | -.02 0.35 -.07 .948 -.002 | -.26 0.42 -.63 .533 -.022 |
| Age | -.44 0.44 -.98 .328 -.051 | -.64 0.52 -1.24 .216 -.064 | .15 0.64 .23 .817 .013 |
| IQ | .03 0.05 .51 .612 .003 | .07 0.06 1.13 .262 .007 | -.02 0.07 -.35 .730 -.002 |
| SDQ-emotional problems D0*SMR  (dummy-coded, reference category) |  |  |  |
| SDQ-emotional problems D0*TBR | -1.49 1.23 -1.21 .227 -.175 | -1.22 1.43 -.85 .395 -.121 | 1.68 2.34 .72 .475 .142 |
| SDQ-emotional problems D0*MPH | -.14 0.64 -.21 .831 -.016 | .12 0.74 .16 .875 .012 | 1.34 .88 1.53 .131 .113 |

*Notes.* ^a^*p*-values less than .05 are highlighted in bold. ^b^Strenghts and Difficulties Questionnaire. ^c^Theta-Beta-ratio. ^d^Methylphenidate.

**Appendix S9.**

*Sensitivity analysis of regression analysis of between-group effects predicting the treatment response based on the severity of SDQ-emotional dysregulation at D0*

| Variable | Clinician | Parents | Teacher |
| --- | --- | --- | --- |
|  | β SE t *p*^a^ *d* | β SE t *p d* | β SE t *p d* |
| SDQ-emotional dysregulation^b^ D0 | -.11 0.26 -.41 .679 -.012 | -.45 0.30 -1.54 .127 -.045 | -.52 0.32 -1.64 .104 -.044 |
| SMR (dummy-coded, reference category) |  |  |  |
| TBR^c^ | 18.23 9.76 1.87 .064 2.140 | 13.75 11.24 1.22 .224 1.360 | 1.88 12.58 .15 .882 .158 |
| MPH^d^ | -3.09 5.92 -.52 .602 -.362 | -2.98 6.81 -.44 .663 -.295 | -8.60 5.60 -1.54 .128 -.725 |
| Gender | -.32 1.93 -.17 .868 -.038 | 1.46 2.22 .66 .512 .145 | 2.49 2.89 .86 .392 .210 |
| Study center | -.36 0.31 -1.16 .250 -.042 | -.21 0.35 -.60 .549 -.021 | -.14 0.40 -.34 .734 -.012 |
| Age | -.44 0.45 -.98 .331 -.051 | **-1.02 0.51 -2.00 .048 -.101** | -.10 0.65 -.15 .878 -.008 |
| IQ | .04 0.05 .77 .441 .005 | .08 0.06 1.43 .156 .008 | -.04 0.07 -.58 .561 -.003 |
| SDQ-emotional dysregulation D0*SMR  (dummy-coded, reference category) |  |  |  |
| SDQ-emotional dysregulation D0*TBR | -1.18 0.64 -1.85 .066 -.138 | -.94 0.73 -1.28 .203 -.093 | -.09 0.93 -.09 .927 -.007 |
| SDQ-emotional dysregulation D0*MPH | -.33 0.37 -.89 .377 .039 | -.42 0.43 -.97 .332 .041 | .04 .44 .08 .934 .003 |

*Notes.* ^a^*p*-values less than .05 are highlighted in bold. ^b^Represents the sum score of the SDQ sub scales emotional problems, conduct problems and hyperactivity. ^c^Theta-Beta-ratio. ^d^Methylphenidate.

**Appendix S10.**

*Correlations between neuropsychological indices and SDQ-emotional problems at D0*

| Variable | BRIEF^a^-total | CPT^b^- omission errors | CPT- commission errors | CPT- hit reaction time (ms) | CPT- variability of hit reaction time (ms) | SDQ^c^-P^d^-emotional problems | SDQ-T^e^-emotionale problems |
| --- | --- | --- | --- | --- | --- | --- | --- |
|  | *r[CI]*^f^  *p*^g^ | *r[CI]*  *p* | *r[CI]*  *p* | *r[CI]*  *p* | *r[CI]*  *p* | *r[CI]*  *p* | *r[CI]*  *p* |
| BRIEF-total | 1 |  |  |  |  |  |  |
| CPT- omission errors | .12[-.05; .28]  .155 | 1 |  |  |  |  |  |
| CPT- commission errors | -.03[-.20; .13]  .687 | -.01[-.17; .15]  .891 | 1 |  |  |  |  |
| CPT- hit reaction Donnetime (ms) | .04[-.12; .21]  .597 | **.49[.35; .60]**  **< .001** | **-.29[-.43; -.13]**  **< .001** | 1 |  |  |  |
| CPT-variability of hit reaction time (ms) | -.15[-.30; .02]  .081 | **-.70[-.77; -.61]**  **< .001** | .09[-.08; .25]  .296 | **-.24[-.39; -.08]**  **.003** | 1 |  |  |
| SDQ-P-emotional problems | **.42[.27; .54]**  **< .001** | .07[-.09; .23]  .398 | -.12[-.28; .04]  .145 | .07[-.09; .23]  .380 | -.11[-.27; .05]  .184 | 1 |  |
| SDQ-T-emotional problems | .13[-.05; .30]  .161 | .09[-.09; .26]  .338 | -.12[-.29; .07]  .209 | -.04[-.22; .14]  .634 | **-.20[-.36; -.02]**  **.030** | **.23[.05; .39]**  **.013** | 1 |

*Notes.* ^a^Behavior Rating Inventory Executive Function. ^b^Conners Continuous Performance Test. ^c^Strenghts and Difficulties Questionnaire. ^d^Parent-rating. ^e^Teacher-rating. ^f^95% confidence interval (two-sided). Estimates are based on Fisher´s r/z transformation. ^g^*p*-values less than .05 are highlighted in bold.

**Appendix S11.**

*Correlations between neuropsychological indices and SDQ-emotional dysregulation at D0*

| Variable | BRIEF^a^-total | CPT^b^- omission errors | CPT- commission errors | CPT- hit reaction time (ms) | CPT- variability of hit reaction time (ms) | SDQ-P^c^-emotional dysregulation^d^ | SDQ-T^e^-emotional dysregulation |
| --- | --- | --- | --- | --- | --- | --- | --- |
|  | *r[CI]*^f^  *p*^g^ | *r[CI]*  *p* | *r[CI]*  *p* | *r[CI]*  *p* | *r[CI]*  *p* | *r[CI]*  *p* | *r[CI]*  *p* |
| BRIEF-total | 1 |  |  |  |  |  |  |
| CPT- omission errors | .12[-.05; .28]  .155 | 1 |  |  |  |  |  |
| CPT- commission errors | -.03[-.20; .13]  .687 | -.01[-.17; .15]  .891 | 1 |  |  |  |  |
| CPT- hit reaction time (ms) | .04[-.12; .21]  .597 | **.49[.35; .60]**  **< .001** | **-.29[-.43; -.13]**  **< .001** | 1 |  |  |  |
| CPT-variability of hit reaction time (ms) | -.15[-.30; .02]  .081 | **-.70[-.77; -.61]**  **< .001** | .09[-.08; .25]  .296 | **-.24[-.39; -.08]**  **.003** | 1 |  |  |
| SDQ-P- emotional dysregulation | **.68[.59; .76]**  **< .001** | .16[-.01; .31]  .059 | -.07[-.23; .10]  .430 | .14[-.03; .29]  .106 | .09[-.08; .25]  .312 | 1 |  |
| SDQ-T- emotional dysregulation | .05[-.13; .23]  .592 | .06[-.12; .24]  .490 | -.04[-.21; .14]  .687 | .01[-.17; .19]  .935 | -.01[-.19; .17]  .896 | .16[-.02; .33]  .079 | 1 |

*Notes.* ^a^Behavior Rating Inventory Executive Function. ^b^Conners Continuous Performance Test. ^c^Parent-rating. ^d^Represents the sum score of the SDQ sub scales emotional problems, conduct problems and hyperactivity. ^e^Teacher-rating. ^f^95% confidence interval (two-sided). Estimates are based on Fisher´s r/z transformation. ^g^*p*-values less than .05 are highlighted in bold.

**Appendix S12.**

*Regression analysis of between-group effects predicting the change in SDQ-emotional problems between D0 and D90 based on neuropsychological indices at D0*

| Variable | Parents | Teacher |
| --- | --- | --- |
|  | β SE t p^a^ d | β SE t p d |
| CPT^b^-hit reaction time (ms) D0 | -.003 0.00 -0.82 .412 -.001 | .006 0.01 1.34 .184 .003 |
| CPT-variability of hit reaction time (ms) D0 | -.003 0.00 -0.61 .540 -.001 | .000 0.00 0.05 .959 .000 |
| CPT-omission errors D0 | .016 0.08 0.20 .842 .006 | -.153 0.08 -1.94 .058 -.064 |
| CPT-commission errors D0 | .003 0.02 0.18 .859 .001 | .019 0.02 0.95 .348 .008 |
| BRIEF^c^-total D0 | .011 0.01 1.28 .204 .004 | -.003 0.01 -0.42 .679 -.001 |
| SMR^d^ (dummy-coded, reference category) |  |  |
| TBR^e^ | -3.250 9.26 -0.35 .726 -1.280 | -9.600 18.20 -0.53 .599 -3.990 |
| MPH^f^ | -.491 3.68 -0.13 .894 -.193 | .034 4.01 0.01 .993 .014 |
| SDQ^g^-emotional problems D0 | **-.512 0.08 -6.80 < .001 -.201** | **-.275 0.07 -3.95 < .001 -.114** |
| Gender | -.070 0.44 -0.16 .874 -.028 | -.019 0.44 -0.04 .966 -.008 |
| Study center | .021 0.07 0.29 .773 .008 | -.113 0.08 -1.46 .151 -.047 |
| Age | .010 0.13 0.08 .934 .004 | .024 0.12 0.19 .848 .010 |
| IQ | -.009 0.01 -0.77 .443 -.004 | .003 0.01 0.25 .800 .001 |
| CPT-hit reaction time (ms) D0*SMR (dummy-coded, reference category) |  |  |
| CPT-hit reaction time (ms) D0*TBR | .004 0.01 0.38 .708 .002 | .004 0.02 0.24 .812 .002 |
| CPT-hit reaction time (ms) D0*MPH | .006 0.01 1.19 .238 .002 | -.001 0.01 -0.08 .935 .000 |
| CPT-variability of hit reaction time (ms) D0*SMR  (dummy-coded, reference category) |  |  |
| CPT-variability of hit reaction time (ms) D0*TBR | -.021 0.02 -1.36 .178 -.008 | -.008 0.02 -0.31 .754 -.003 |
| CPT-variability of hit reaction time (ms) D0*MPH | -.006 0.01 -0.90 .368 -.002 | -.004 0.01 -0.58 .564 -.002 |
| CPT-omission errors D0*SMR (dummy-coded, reference category) |  |  |
| CPT-omission errors D0*TBR | -.086 0.15 0.59 .556 .034 | .038 0.16 0.24 .810 .016 |
| CPT-omission errors D0*MPH | -.001 0.10 0.01 .995 -.000 | .165 0.09 1.77 .082 .069 |
| CPT-commission errors D0*SMR (dummy-coded, reference category) |  |  |
| CPT-commission errors D0*TBR | .045 0.06 0.79 .431 .018 | .063 0.12 0.54 .593 .026 |
| CPT-commission errors D0*MPH | .007 0.02 0.29 .775 .003 | -.021 0.02 -0.86 .392 -.009 |
| BRIEF-total D0*SMR  (dummy-coded, reference category) |  |  |
| BRIEF-total D0*TBR | .004 0.03 0.16 .874 .002 | .019 0.04 0.55 .584 .008 |
| BRIEF-total D0*MPH | -.012 0.01 -0.93 .353 -.005 | .004 0.01 0.34 .733 .002 |

*Notes.* ^a^*p*-values less than .05 are highlighted in bold. ^b^Conners Continuous Performance Test. ^c^Behavior Rating Inventory Executive Function. ^d^Sensorimotor rhythm. ^e^Theta-Beta-ratio. ^f^Methylphenidate. ^g^Strenghts and Difficulties Questionnaire.

**Appendix S13.**

*Regression analysis of between-group effects predicting the change in SDQ-emotional dysregulation between D0 and D90 based on neuropsychological indices at D0*

| Variable | Parents | Teacher |
| --- | --- | --- |
|  | β SE t p^a^ d | β SE t p d |
| CPT^b^-hit reaction time (ms) D0 | .005 0.01 0.69 .491 .001 | .005 0.01 0.44 .661 .001 |
| CPT-variability of hit reaction time (ms) D0 | -.004 0.01 -0.53 .595 -.001 | .010 0.01 1.00 .319 .002 |
| CPT-omission errors D0 | -.046 0.13 -0.34 .729 -.010 | -.274 0.18 -1.50 .138 -.055 |
| CPT-commission errors D0 | .007 0.03 0.26 .795 .002 | .033 0.05 0.69 .492 .007 |
| BRIEF^c^-total D0 | .015 0.02 1.01 .315 .003 | .009 0.02 0.54 .589 .002 |
| SMR^d^ (dummy-coded, reference category) |  |  |
| TBR^e^ | -2.955 14.97 -0.20 .844 -.665 | -14.625 42.62 -0.34 .733 -2.940 |
| MPH^f^ | 1.909 6.11 0.31 .755 .429 | 7.706 9.44 0.82 .418 1.550 |
| SDQ-emotional dysregulation^g^ D0 | **-.478 0.09 -5.21 < .001 -.108** | **-.322 0.08 -3.85 < .001 -.065** |
| Gender | .516 0.71 0.72 .472 .116 | .693 1.04 0.67 .509 .139 |
| Study center | -.069 0.12 -0.59 .558 -.016 | -.061 0.18 -0.35 .731 -.012 |
| Age | -.196 0.21 -0.95 .345 -.044 | -.293 0.30 -0.99 .324 -.059 |
| IQ | .013 0.02 0.67 .504 .003 | .023 0.03 0.83 .410 .005 |
| CPT-hit reaction time (ms) D0*SMR (dummy-coded, reference category) |  |  |
| CPT-hit reaction time (ms) D0*TBR | .003 0.02 0.16 .877 .001 | .015 0.04 0.34 .732 .003 |
| CPT-hit reaction time (ms) D0*MPH | -.002 0.01 -0.25 .802 -.001 | -.004 0.01 -0.28 .782 -.001 |
| CPT-variability of hit reaction time (ms) D0*SMR  (dummy-coded, reference category) |  |  |
| CPT-variability of hit reaction time (ms) D0*TBR | -.045 0.03 -1.82 .072 -.010 | -.032 0.06 -0.58 .567 -.007 |
| CPT-variability of hit reaction time (ms) D0*MPH | .003 0.01 0.26 .794 .001 | -.016 0.02 -1.00 .320 -.003 |
| CPT-omission errors D0*SMR (dummy-coded, reference category) |  |  |
| CPT-omission errors D0*TBR | .435 0.23 1.88 .063 .098 | .549 0.37 1.49 .143 .010 |
| CPT-omission errors D0*MPH | .142 0.16 0.90 .373 .032 | .379 0.21 1.77 .082 .076 |
| CPT-commission errors D0*SMR (dummy-coded, reference category) |  |  |
| CPT-commission errors D0*TBR | .053 0.09 0.58 .567 .012 | -.010 0.27 -0.04 .972 -.002 |
| CPT-commission errors D0*MPH | -.001 0.04 -0.22 .983 -.000 | -.085 0.06 -1.52 .135 -.017 |
| BRIEF-total D0*SMR  (dummy-coded, reference category) |  |  |
| BRIEF-total D0*TBR | .009 0.04 0.23 .818 .002 | .029 0.08 0.36 .722 .006 |
| BRIEF-total D0*MPH | -.022 0.02 -1.05 .298 -.005 | -.004 0.03 -0.80 .426 -.005 |

*Notes.* ^a^*p*-values less than .05 are highlighted in bold. ^b^Conners Continuous Performance Test. ^c^Behavior Rating Inventory Executive Function. ^d^Sensorimotor rhythm. ^e^Theta-Beta-ratio. ^f^Methylphenidate. ^g^Represents the sum score of the SDQ sub scales emotional problems, conduct problems and hyperactivity.

**Appendix S14.**

*Sensitivity analysis of regression analysis of between-group effects predicting the change in SDQ-emotional problems between D0 and D90 based on neuropsychological indices at D0*

| Variable | Parents | Teacher |
| --- | --- | --- |
|  | β SE t p^a^ d | β SE t p d |
| CPT^b^-hit reaction time (ms)D0 | -.004 0.01 -.89 .375 -.002 | **.010 0.01 1.92 .059 .004** |
| CPT-variability of hit reaction time (ms) D0 | -.004 0.01 -.82 .416 -.002 | .001 0.01 .26 .797 .001 |
| CPT-omission errors D0 | .031 0.10 .32 .752 .012 | **-.215 0.09 -2.49 .016 -.089** |
| CPT-commission errors D0 | .015 0.02 .76 .447 .006 | .033 0.02 1.47 .148 .014 |
| BRIEF^c^-total D0 | -.010 0.01 -1.11 .268 -.004 | -.010 0.01 -1.21 .229 -.004 |
| SMR^d^ (dummy-coded, reference category) |  |  |
| TBR^e^ | 3.720 10.90 .34 .733 1.460 | -7.800 20.20 -.39 .700 -3.240 |
| MPH^f^ | -1.250 4.36 -.29 .774 -.492 | -.484 4.46 -.11 .914 -.201 |
| Gender | .183 0.52 .35 .726 .072 | .068 0.49 .14 .889 .028 |
| Study center | .024 0.09 .28 .779 .009 | **-.193 0.08 -2.32 .024 -.080** |
| Age | .047 0.15 .32 .752 .018 | .048 0.14 .35 .728 .020 |
| IQ | -.014 0.01 -1.03 .304 -.006 | .010 0.01 .74 .462 .004 |
| CPT-hit reaction time (ms) D0*SMR  (dummy-coded, reference category) |  |  |
| CPT-hit reaction time (ms) D0*TBR | -.001 0.01 -.09 .928 .000 | -.001 0.02 -.03 .979 .000 |
| CPT-hit reaction time (ms) D0*MPH | .006 0.01 1.04 .299 .002 | -.002 0.01 -.23 .821 -.001 |
| CPT-variability of hit reaction time (ms) D0*SMR  (dummy-coded, reference category) |  |  |
| CPT-variability of hit reaction time (ms) D0*TBR | -.015 0.02 -.81 .421 -.006 | -.005 0.03 -.17 .866 -.002 |
| CPT-variability of hit reaction time (ms) D0*MPH | -.004 0.01 -.47 .639 -.002 | -.008 0.01 -1.10 .274 -.004 |
| CPT-omission errors D0*SMR  (dummy-coded, reference category) |  |  |
| CPT-omission errors D0*TBR | .032 0.17 .19 .851 .013 | .071 0.18 .41 .686 .030 |
| CPT-omission errors D0*MPH | -.003 0.12 -.02 .982 -.001 | **.257 0.10 2.54 .014 .107** |
| CPT-commission errors D0*SMR  (dummy-coded, reference category) |  |  |
| CPT-commission errors D0*TBR | .013 0.07 .20 .844 .005 | .041 0.13 .32 .750 .017 |
| CPT-commission errors D0*MPH | -.006 0.03 -.23 .820 -.002 | -.027 0.03 -1.00 .321 -.011 |
| BRIEF-total D0*SMR (dummy-coded, reference category) |  |  |
| BRIEF-total D0*TBR | -.009 0.03 -.32 .751 -.004 | .025 0.04 .64 .524 .010 |
| BRIEF-total D0*MPH | -.006 0.02 -.37 .715 -.002 | .011 0.01 .79 .434 .004 |

*Notes.* ^a^*p*-values less than .05 are highlighted in bold. ^b^Conners Continuous Performance Test. ^c^Behavior Rating Inventory Executive Function. ^d^Sensorimotor rhythm. ^e^Theta-Beta-ration. ^f^Methylphenidate.

**Appendix S15.**

*Sensitivity analysis of regression analysis of between-group effects predicting the change in SDQ-emotional dysregulation between D0 and D90 based on neuropsychological indices at D0*

| Variable | Parents | Teacher |
| --- | --- | --- |
|  | β SE t p^a^ d | β SE t p d |
| CPT^b^-hit reaction time (ms)D0 | .007 0.01 .92 .359 .002 | .007 0.01 .62 .539 .002 |
| CPT-variability of hit reaction time (ms) D0 | -.005 0.01 -.60 .550 -.001 | .011 0.01 1.02 .312 .002 |
| CPT-omission errors D0 | -.109 0.15 -.74 .459 -.025 | -.267 0.20 -1.32 .191 -.054 |
| CPT-commission errors D0 | .027 0.03 .89 .376 .006 | .029 0.05 .56 .580 .006 |
| BRIEF^c^-total D0 | -.028 0.01 -1.94 .055 -.006 | .017 1.16 .02 .988 .000 |
| SMR^d^ (dummy-coded, reference category) |  |  |
| TBR^e^ | 8.711 16.52 .53 .599 1.960 | -10.735 47.12 -.23 .821 -2.160 |
| MPH^f^ | 5.574 6.77 .82 .412 1.250 | 4.706 10.41 .45 .653 .946 |
| Gender | .973 0.79 1.23 .221 .219 | .017 1.14 .02 .988 .003 |
| Study center | .001 0.13 .07 .941 .002 | -.133 0.19 -.69 .494 -.027 |
| Age | -.017 0.23 -.07 .942 -.004 | -.073 0.32 -.23 .819 -.015 |
| IQ | -.007 0.02 -.31 .756 -.002 | .037 0.03 1.21 .232 .008 |
| CPT-hit reaction time (ms) D0*SMR  (dummy-coded, reference category) |  |  |
| CPT-hit reaction time (ms) D0*TBR | -.007 0.02 -.39 .695 -.002 | .006 0.05 .12 .902 .001 |
| CPT-hit reaction time (ms) D0*MPH | -.009 0.01 -.93 .356 -.002 | -.004 0.02 -.28 .782 -.001 |
| CPT-variability of hit reaction time (ms) D0*SMR  (dummy-coded, reference category) |  |  |
| CPT-variability of hit reaction time (ms) D0*TBR | -.036 0.03 -1.31 .195 -.008 | -.011 0.06 -.18 .861 -.002 |
| CPT-variability of hit reaction time (ms) D0*MPH | .002 0.01 .15 .883 .000 | -.020 0.02 -1.15 .254 -.004 |
| CPT-omission errors D0*SMR  (dummy-coded, reference category) |  |  |
| CPT-omission errors D0*TBR | .455 0.26 1.76 .081 .102 | .455 0.41 1.12 .269 .091 |
| CPT-omission errors D0*MPH | .199 0.18 1.23 .262 .045 | .459 0.24 1.95 .056 .092 |
| CPT-commission errors D0*SMR  (dummy-coded, reference category) |  |  |
| CPT-commission errors D0*TBR | .014 0.10 .13 .894 .003 | -.009 0.30 -.03 .977 -.002 |
| CPT-commission errors D0*MPH | -.011 0.04 -.26 .795 -.002 | -.057 0.06 -.93 .358 -.012 |
| BRIEF-total D0*SMR (dummy-coded, reference category) |  |  |
| BRIEF-total D0*TBR | -.019 0.04 -.42 .673 -.004 | .022 0.09 .24 .813 .004 |
| BRIEF-total D0*MPH | -.024 0.02 -1.05 .295 -.006 | -.015 0.03 -.47 .642 -.003 |

*Notes.* ^a^*p*-values less than .05 are highlighted in bold. ^b^Conners Continuous Performance Test. ^c^Behavior Rating Inventory Executive Function. ^d^Sensorimotor rhythm. ^e^Theta-Beta-ration. ^f^Methylphenidate
